# Dysregulated Microbiota-Driven Gasdermin D Activation Promotes Colitis Development by Mediating IL-18 Release

**DOI:** 10.3389/fimmu.2021.750841

**Published:** 2021-10-14

**Authors:** Hanchao Gao, Mengtao Cao, Yikun Yao, Wenjun Hu, Huimin Sun, Yingwei Zhang, Changchun Zeng, Jia Tang, Shaodong Luan, Pengfei Chen

**Affiliations:** ^1^ Department of Medical Laboratory, Shenzhen Longhua District Central Hospital, Affiliated Central Hospital of Shenzhen Longhua District, Guangdong Medical University, Shenzhen, China; ^2^ Molecular Development of the Immune System Section, Laboratory of Immune System Biology, and Clinical Genomics Program, National Institute of Allergy and Infectious Diseases (NIAID), National Institutes of Health, Bethesda, MD, United States; ^3^ Department of Anesthesiology, 305 Hospital of People’s Liberation Army of China (PLA), Beijing, China; ^4^ National Health Commission (NHC), Key Laboratory of Male Reproduction and Genetics, Guangdong Provincial Reproductive Science Institute (Guangdong Provincial Fertility Hospital), Guangzhou, China

**Keywords:** gut microbiota, gasdermin D, colitis, intestinal epithelial cell, IL-18

## Abstract

The balance between gut microbiota and host is critical for maintaining host health. Although dysregulation of the gut microbiota triggers the development of various inflammatory diseases, including colitis, the molecular mechanism of microbiota-driven colitis development is largely unknown. Here, we found that gasdermin D (GSDMD) was activated during acute colitis. In the dextran sulfate sodium (DSS)-induced colitis model, compared to wild-type mice, *Gsdmd*-deficient mice had less colitis severity. Mechanistically, GSDMD expression in intestinal epithelial cells (IECs), but not infiltrating immune cells, was critical for GSDMD-mediated colitis progression. Moreover, commensal *Escherichia coli* (*E. coli*) largely overgrew during colitis, and then the dysregulated commensal *E. coli* mediated GSDMD activation. Furthermore, the activated GSDMD promoted the release of interleukin-18 (IL-18), but not the transcript or maturation level of IL-18, which in turn mediated goblet cell loss to induce colitis development. Thus, GSDMD promotes colitis development by mediating IL-18 release, and the microbiota can mediate colitis pathogenesis through regulation of GSDMD activation. Our results provide a potential molecular mechanism by which the microbiota-driven GSDMD activation contributes to colitis pathogenesis.

## Introduction

The gut microbiota constantly affects host nutrient absorption and immune system development (1, 2). The mutual relationship between the gut microbiota and host is crucial for maintaining intestinal homeostasis (3–5). When this homeostatic balance is compromised, excessive immune responses are triggered and consequently contribute to various inflammatory diseases, including inflammatory bowel disease (IBD) and colorectal cancer (4, 6–8). Various reports have shown that the dysregulation of gut microbiota and the host immune system is critical for IBD development (3, 4, 9). However, it is still largely unknown how the dysregulated microbiota promotes IBD development.

IBD, including ulcerative colitis (UC) and Crohn’s disease (CD), is a chronic inflammatory disease of the gastrointestinal tract characterized by abdominal pain, diarrhea, bloody stools and body weight loss (8, 10). Dextran sulfate sodium (DSS)-induced colitis is a generally used mouse model to mimic human UC (11). The inflammasome is an intracellular multiprotein complex that induces mature interleukin-1β (IL-1β) and IL-18 production and mediates cell pyroptosis (12). It has been reported that the inflammasome pathway is involved in IBD development (13). The major components and effector molecules of the inflammasome, such as NLRP3, ASC, AIM2, Caspase 1, Caspase 11, IL-18, and IL-1β, play important roles in DSS-induced colitis. However, controversial roles of some of these genes in colitis have been observed by different studies (14–27).

Recently, GSDMD was identified as another novel inflammasome effector that mediates cell pyroptosis by forming membrane pores (28, 29). When the canonical or noncanonical inflammasome is induced, GSDMD is activated and cleaved into the N-terminal domain (GSDMD-N) and C-terminal domain (GSDMD-C) by Caspase 1 and Caspase 11 in mice or Caspase 4/5 in humans. Inflammasome components, such as NLRP1, NLRP2, NLRP3, NLRP6, NLRC4 and NLRP9b, could activate Caspase 1, which in turn activated GSDMD to mediate canonical pyroptosis (30). In noncanonical infalmmasome, lipopolysaccharide (LPS) directly bound and activated Caspase 4/5/11, and then the activated Caspase 4/5/11 cleaved GSDMD to initiate pyroptosis. GSDMD-N, which is the active form of GSDMD, forms membrane pores to mediate mature IL-1β and IL-18 secretion as well as cell pyroptosis, while GSDMD-C has the opposite suppressive effect by binding GSDMD-N (28, 31, 32).

Several studies have reported the role of GSDMD in neuron system. GSDMD mediated cell pyroptosis and promoted neuroinflammation in many nervous system diseases, including Parkinson’s Disease (PD), Multiple Sclerosis (MS), spinal cord injury, stroke, Traumatic Brain injury (TBI) and Zika virus-induced brain atrophy (30, 33). *Gsdmd* deficient mice showed impaired neuroinflammation and pathogenesis of experimental autoimmune encephalomyelitis (EAE), a well-characterized animal model of MS (34). Liu et al, showed that ablation of Caspase 1 decreased TBI-induced pyroptosis (35). Baicalein, a flavonoid isolated from the traditional Chinese medicinal herbal *Scutellaria baicalensis Georgi*, reduced neuroinflammation in MPTP-induced PD mice through suppressing NLRP3/Caspase-1/GSDMD Pathway (36). Moreover, other studies suggested that GSDMD might be a promising target for stroke therapy (37, 38). These researches indicate that GSDMD mediated-pyroptosis have played a critical role in neuroinflammation and nervous system diseases. Although GSDMD is a critical pro-inflammatory gene in neuron system, the *in vivo* role of GSDMD in intestinal inflammation remains unclear. Herein, our study found that dysregulated microbiota activated GSDMD, which in turn mediated DSS-induced colitis development by promoting IL-18 release.

## Material and Methods

### Reagent and Cell Lines

Anti-GSDMD and anti-IL-18 were purchased from Abcam (Cambridge, UK). Anti-GAPDH, anti-cleaved Caspase 3, and anti-Caspase 3 were purchased from Cell Signaling Technology (Danvers, MA, US). Anti-Caspase 11 was purchased from Novus (Littleton, CO, USA). Anti-Caspase 1(p20) was purchased from AdipoGen (San Diego, CA, USA). Anti-HA was from Biolegend (San Diego, CA, USA). HT-29 cells were maintained in DMEM containing 10% (vol/vol) FBS, penicillin (100 U/ml) and streptomycin (100 μg/ml).

### Mice

*Gsdmd^-/-^* mice on the C57BL/6 background were purchased from GemPharmatech (Nanjing, China). All mice were maintained in specific pathogen-free conditions and littermates from the same mouse line were bred as strict controls. For the cohousing assay, 3-week old sex-matched wild-type (WT) and *Gsdmd^-/-^* mice were cohoused at a 1:1 ratio for 4 weeks before exposure to DSS and left together during colitis. All animal experiments were performed in compliance with the guide for the care and use of laboratory animals and were approved by the institutional biomedical research ethics committee of Guangdong Medical University.

### Induction of Colitis

Six to eight-week-old WT and *Gsdmd^-/-^* mice (n=5 or 6/group) were given with 3% DSS (Meilunbio, Dalian, China) in drinking water for 5 days, and then followed by normal drinking water until Day 8. Mice were sacrificed for tissue analyses on Day 8. For survival analysis, mice (n=10) were given 3.5% DSS solution in drinking water for 5 days followed by normal drinking water until Day 15.

### Determination of Clinical Scores

After the DSS challenge, fresh stool samples were collected on Day 6, and scoring for stool consistency and occult blood was performed as previously described (7, 8). In brief, stool scores were determined as follows: 0, well-formed pellets; 1, semiformed stools that did not adhere to the anus; 2, semiformed stools that adhered to the anus; and 3, liquid stools that adhered to the anus. Bleeding scores were determined as follows: 0, no blood by using hemoccult; 1, positive hemoccult; 2, blood traces in stool visible; 3, gross rectal bleeding.

### Determination of Cell Proliferation and Death

For determination of cell proliferation, paraffin sections were stained using anti-Ki67 antibody (Ebioscience, San Diego, CA, USA). For determination of cell death, a TUNEL assay was performed with the *In situ* Cell Death Kit (Roche, Mannheim, Germany) according to the manufacturer’s recommendations. The positive cells were counted by light microscopy.

### Bone Marrow Chimeras

Bone marrow transfer was used to create *Gsdmd^-/-^* chimera mice wherein the genetic deficiency of *Gsdmd* was confined to either circulating cells (*Gsdmd^-/-^* →WT chimera) or nonhematopoietic tissue (WT→*Gsdmd^-/-^*). In brief, 6 to 8 week-old recipient WT control mice or *Gsdmd^-/-^* mice were lethally irradiated with 800 cGy, and then the recipient WT control mice or *Gsdmd^-/-^* mice were injected in the tail vein with 5×10^6^-1×10^7^ mixed bone marrow cells from donor WT mice or *Gsdmd^-/-^* mice. Four chimera groups were generated: WT→WT (n=6), WT→*Gsdmd^-/-^* (n=8), *Gsdmd^-/-^* →WT (n=6), and *Gsdmd^-/-^* →*Gsdmd^-/-^* (n=7). The transplanted mice were given drinking water containing 2 g/L neomycin sulfate (Meilunbio) for 2 weeks. After 8 weeks of bone marrow reconstitution, colitis was induced in the mice with 2.5% DSS for 5 days, followed by normal drinking water until Day 8. Mice were sacrificed for tissue analyses on Day 8.

### Isolation of Intestinal Epithelial Cells and Lamina Propria Lymphocytes

Isolation of intestinal epithelial cells (IECs) and lamina propria lymphocytes (LPLs) was previously described (7, 8). Briefly, the dissected colon tissues were washed in HBSS buffer and then cut into pieces and digested by 75 U/ml collagenase type XI (Sigma, St. Louis, MO, USA) and 20 μg/ml dispase (Sigma) in DMEM supplemented with 1% (vol/vol) FBS, penicillin (100 U/ml) and streptomycin (100 μg/ml) at 37°C. After 3 hours of digestion, crypts containing IECs were isolated from the supernatant of the digestion buffer by centrifugation at 300 g for 5 min. The isolated crypts were analyzed by immunoblot or RT-PCR.

For isolation of LPLs, the dissected colon tissues were washed in HBSS buffer, and the epithelium was removed by shaking at 250 rpm shaking at 37°C in HBSS buffer containing 30 mM EDTA, 1 mM DTT and 5% FBS for 30 min. After sedimentation, the crypts containing supernatant were discarded and the remaining colon tissues were further cut into small pieces and digested by 200 U/ml collagenase type VIII (Sigma) and 150 μg/ml DNase I (Sigma) in RPMI-1640 medium supplemented with 5% (vol/vol) FBS, penicillin (100 U/ml) and streptomycin (100 μg/ml) at 37°C for 1 hour. The supernatant was centrifuged at 450 g for 5 min to collect cells and the LPLs were further isolated by Percoll (40%/80%; Solarbio, Beijing, China). The isolated LPLs were then subjected to western-blotting or RT-PCR analyses.

### Adenovirus-Mediated GSDMD-C Expression in Mice

Mouse GSDMD-C with an HA tag was cloned into the pAdTrack-CMV vector and then recombined with the pAdEasy-1 vector. Recombinant Adv-GSDMD-C or empty vector (Adv-EV) was transfected into HEK 293A cells. Viruses were packaged and amplified as described (39). After titration, 2×10^10^ adenovirus particles were intraperitoneally injected into the indicated mice every other day. Four days later, colitis was induced in the mice with DSS solution as described.

### Recombinant Mouse IL-1β or IL-18

Recombinant mouse IL-1β (Novoprotein, Shanghai, China) or PBS as negative control was injected into *Gsdmd^-/-^* mice intraperitoneally at a concentration of 0.5 μg or 1 μg per mouse in 200 µl sterile PBS on Days 1, 2, 3, 4, 5, and 6 during DSS-induced colitis.

Recombinant mouse IL-18 (Novoprotein) or PBS as negative control was injected into *Gsdmd^-/-^* mice intraperitoneally at a concentration of 1 μg per mouse in 200 µl sterile PBS from Day 1 to Day 8 during DSS-induced colitis.

### Commensal Depletion

The mice were treated with a cocktail of antibiotics in drinking water as previously reported (7). Briefly, mice were treated with of 1 mg/ml neomycin, 0.5 mg/ml vancomycin, 1 mg/ml metronidazole and 1 mg/ml ampicillin for 4 weeks. Every week fresh antibiotics were supplied. After 4 weeks, drinking water was further supplemented with 1 mg/ml streptomycin, 170 μg/ml gentamicin, 125 μg/ml ciprofloxacin, and 1 mg/ml bacitracin for one week. More than 99.9% of intestinal microbes were removed by this method. After mice were treated with antibiotics for 5 weeks, colitis was induced in the indicated mice as described in the section on the induction of colitis. For *E. coli-*induced GSDMD activation *in vivo*, antibiotic-treated mice were treated with 3% DSS for 5 days, during which at Day 4 and Day 5 the mice were given 10^10^ CFU of *E. coli* isolated from eosin-methylene blue (EMB) agar plates by gavage and rectal administration. The mice were sacrificed on Day 6, and colon tissues were obtained for further analysis.

### LPS Transfection and Cell Viability Measurement

LPS was electroporated into HT-29 cells using the Neon Transfection System (Thermo Fisher Scientific, Waltham, MA, USA) following the manufacturer’s instructions as previously reported (28, 40). Briefly, 1×10^6^ HT-29 cells were transfected with 1 μg LPS. Cell viability was measured at 2.5 hours after LPS transfection. Cell viability was determined by the CellTiter-Glo Luminescent Cell Viability Assay (Promega, Madison, WI, USA).

### RT–PCR

Real-time PCR has been reported previously (41). Briefly, total RNA of cells or tissue was extracted with TRIzol^®^ Reagent (Invitrogen, Shanghai, China). cDNA samples were synthesized with PrimeScript™ RT Master Mix (Takara Bio, Dalian, China). The levels of genes of the interest were quantified using TB Green^®^ *Premix Ex Taq* ^™^ (Tli RNaseH Plus) (Takara Bio). The expression levels of the genes were calculated by the 2^-ΔΔCt^ method and normalized to β-actin. Amplification of cDNA was performed using a ViiA 7 Real-Time PCR system (Applied Biosystems, Foster City, CA, USA) with the sequences of oligonucleotide primers shown in **Supplementary Table 1**.

### Isolation of Bacterial Genomic DNA and Microbiota Analysis by Quantitative PCR

Fresh fecal pellets were obtained at the indicated time points. Bacterial genomic DNA was extracted with the TIANamp Stool DNA Kit (Tiangen Biotech, Beijing, China) with the optional high-temperature step (90°C) directly. To assess the abundance of specific intestinal bacterial groups, the extracted bacterial genomic DNA was analyzed with TB Green^®^ Premix Ex Taq™ (Tli RNaseH Plus) (Takara Bio) on a ViiA 7 Real-Time PCR System. Signals were normalized to universal bacteria, and normalized data were used to calculate relative levels of 16S rRNA gene expression of indicated bacterial groups. The 16S rDNA primer sequences are shown in **Supplementary Table 2**.

### Immunoblot Analysis

The procedure of immunoblot analysis has been previously reported (42). In brief, the cells or tissues were lysed for 30 min in ice-cold RIPA lysis buffer supplemented with 10 mM sodium fluoride (NaF), 1 mM Na_3_VO_4_, 1 mM phenylmethylsulfonyl fluoride, and a complete protease inhibitor cocktail (Roche) and separated by SDS–PAGE. After the proteins were transferred onto polyvinylidene fluoride (PVDF) membranes (Millipore, Darmstadt, Germany), the PVDF membranes were blocked at room temperature for 1 hour and then incubated with primary antibody overnight at 4°C. After incubation with secondary antibody for 1 hour at room temperature, the blots were visualized with enhanced chemiluminescence (ECL) detection reagents (Millipore).

### Histology and Immunostaining

The colon tissue was fixed in 4% paraformaldehyde (PFA) for 48 hours and then embedded in paraffin wax. For assessment of injury, 5-mm sections were stained with hematoxylin-eosin (H&E). Alcian blue (AB)/periodic acid-Schiff (PAS) staining was used to assess goblet cells in the colon. To assess macrophage infiltration, sections were stained with rat anti-mouse F4/80 antibody (Abcam) followed by goat anti-rat biotin conjugate. After incubation with ABC reagent, stained sections were photographed by light microscopy.

### Enzyme-Linked Immunosorbent Assay

To measure IL-1β and IL-18 in colon tissues, a part of the colon was weighed and homogenized mechanically in PBS containing 1% NP-40 and a complete protease inhibitor cocktail (Roche). The protein level of IL-1β or IL-18 in colon homogenate was measured with ELISA kits (R&D Systems, Minneapolis, MN; Sino Biological, Beijing, China) according to the manufacturer’s instructions.

The procedure of measuring the cytokines from cultured colon explants has been described previously (21). In brief, colon tissue explants were obtained and rinsed with PBS three times and cultured for 24 hours in DMEM containing 10% FBS, L-glutamine, 1% P/S and neomycin at 37°C. The protein level of IL-1β or IL-18 in the supernatant of cultured colon tissue was measured with ELISA kits (R&D Systems; Sino Biological) according to the manufacturer’s instructions.

### Statistical Analysis

Prism software (GraphPad Software) was used to perform statistical analysis and graph development. A two-tailed Student’s t test was used to compare differences between two groups. Survival curves are presented using the Kaplan–Meier method, and significance was calculated by the log-rank (Mantel-Cox) test. *p* values < 0.05 were considered significant.

## Results

### GSDMD Is Activated in DSS-Induced Colitis

**Figure 1:**
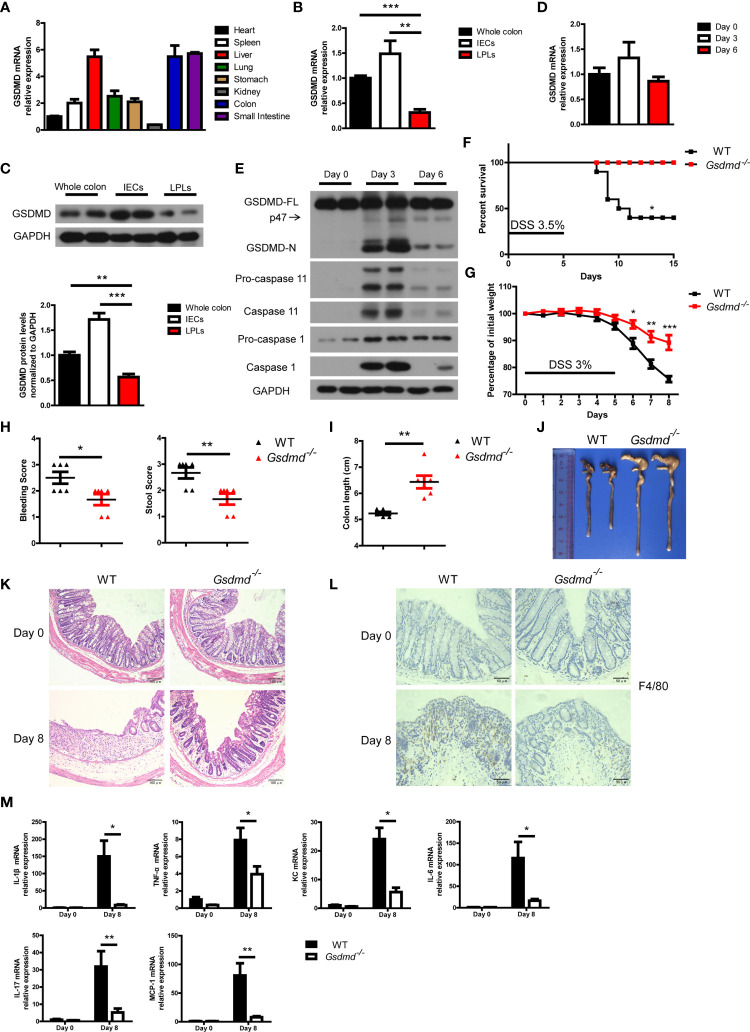
DSS-induced colitis is ameliorated in *Gsdmd*-deficient mice. **(A)** Quantitative mRNA expression of GSDMD in different C57BL/6 wild-type (WT) mouse tissues as indicated (n = 4). **(B)** Quantitative mRNA expression of GSDMD from WT mouse whole colon, IECs, or LPLs (n = 4). **(C)** Immunoblot analysis of GSDMD expression in WT mouse whole colon, IECs, or LPLs. The lower panel shows the quantitative analysis of GSDMD protein levels (n = 4). **(D)** Quantitative mRNA expression of GSDMD in WT mouse colons at the indicated times during DSS-induced colitis. **(E)** Immunoblot analysis of the expression of the indicated genes in the whole colon of WT mice at the indicated times during DSS-induced colitis. **(F)** Survival analysis of WT (n = 10) or *Gsdmd^-/-^* (n = 10) mice treated with 3.5% DSS for 5 days. **(G)** Body weight change of WT (n = 6) or *Gsdmd^-/-^* (n = 6) mice during the progression of DSS-induced colitis. **(H)** Bleeding score and stool score of the WT (n = 6) or *Gsdmd^-/-^* (n = 6) mice on Day 6 of the colitis model as in **(G)**. **(I, J)** Colon length **(I)** and the macroscopic view **(J)** of WT or *Gsdmd^-/-^* mouse colons on Day 8 of the colitis model as in **(G)**. **(K)** Hematoxylin and eosin (H&E) staining of the representative colons from the mice on Day 0 or Day 8 of the colitis model as in **(G)** (200× magnification). **(L)** F4/80 staining of the representative colons from the WT or *Gsdmd^-/-^* mice on Day 0 or Day 8 of the colitis model as in **(G)** (400× magnification). **(M)** Quantitative mRNA expression of inflammatory genes as indicated from the colon of WT or *Gsdmd^-/-^* mice on Day 0 or Day 8 of the colitis model as in **(G)**. Data are representative of three **(E–M)** or four **(A–D)** independent experiments (mean ± SEM in **(A–D)**, **(G–I)** and **(M)**. *p < 0.05, **p < 0.01, ***p < 0.001 by Student’s t test.

Mouse GSDMD was ubiquitously expressed in the examined tissues and was highly expressed in the liver, colon and small intestine (**Figure 1A**). We isolated intestinal epithelial cells (IECs) and lamina propria lymphocytes (LPLs) from mouse colon tissues, and we found that both the mRNA and protein levels of GSDMD in IECs were much higher than those of GSDMD in LPLs (**Figures 1B, C** **)**, suggesting that GSDMD is mainly expressed in IECs. Although the mRNA or protein level of full length GSDMD (GSDMD-FL) was not significantly regulated in the DSS-induced colitis model (**Figures 1D, E** **)**, GSDMD was strongly cleaved to activated GSDMD-N form (p30 fragment) on Day 3 and the activation decreased on Day 6 during DSS-induced colitis (**Figure 1E**). In addition to GSDMD-N, another p47 fragment was also cleaved during DSS-induced colitis (**Figure 1E**). We also found that the expression of Caspase 1 or Caspase 11 was dramatically induced on Day 3 in the DSS-induced colitis model (**Figure 1E** **and** **Supplementary Figure 1**). These data suggest that GSDMD is activated in the experimental colitis model.

### GSDMD Aggravates the Pathology of DSS-Induced Colitis

Next, we asked whether GSDMD plays an important role in colitis development. To this end, *Gsdmd*-deficient mice, as well as their control mice, were assessed with the DSS-induced colitis model. We found that, after challenging these mice with 3.5% DSS for 5 days, 60% of the WT littermate mice died within 15 days, while all *Gsdmd^-/-^* mice survived (**Figure 1F**). Treating the mice with 3% DSS for 5 days led to significant body weight loss in WT control mice, while the body weight loss was alleviated in *Gsdmd*-deficient mice (**Figure 1G**). The bleeding score and stool score, which indicate colitis severity, were much lower in *Gsdmd^-/-^* mice (**Figure 1H**), and consistently, the colon lengths were longer in *Gsdmd^-/-^* mice (**Figures 1I, J** **)**. The pathology of the injured colons from WT mice was more severe than that of *Gsdmd^-/-^* mice as indicated by hematoxylin-eosin staining showing the structure of the colon (**Figure 1K**). Moreover, macrophage infiltration was remarkably reduced in *Gsdmd^-/-^* mice compared to WT mice (**Figure 1L**). The mRNA levels of inflammatory cytokines, such as IL-1β, TNF-α, KC, IL-6, IL-17, and MCP-1, were largely upregulated in WT mice, while the expression of these genes was only minor induced in *Gsdmd^-/-^* mice (**Figure 1M**). These data indicate that GSDMD is essential for DSS-induced colitis.

Moreover, to determine whether GSDMD affects IEC proliferation or IEC death during colitis, we analyzed intestinal cell proliferation and cell death by Ki67 and TUNEL staining, respectively. GSDMD deficiency reduced IEC proliferation and IEC death in DSS-induced colitis (**Supplementary Figures 2A-D**). GSDMD is the executor of cell pyroptosis, and TUNEL staining showed that cell death was reduced in *Gsdmd^-/-^* mice (**Supplementary Figures 2C, D** **)**. To discriminate cell apoptosis and pyroptosis, we checked cleaved Caspase 3, a marker of cell apoptosis, and found that the level of cleaved Caspase 3 did not change between WT and *Gsdmd^-/-^* mice in DSS-induced colitis (**Supplementary Figure 2E**). These data show that GSDMD is most likely to mediate IEC pyroptosis during colitis.

Because antimicrobial peptides (AMPs) are critical in maintaining mucosal barrier integrity and suppressing colitis development, we investigated whether GSDMD promotes colitis by decreasing AMP production. We found that the mRNA levels of AMPs largely increased in WT mice treated with DSS. We found that the mRNA levels of AMPs in these mice were much higher than those in DSS-treated *Gsdmd^-/-^* mice (**Supplementary Figure 3**). The data suggest that AMPs are not the major factor causing GSDMD to promote colitis development.

### GSDMD Expression in Intestinal Epithelial Cells Is Critical for the Progression of Colitis

**Figure 2:**
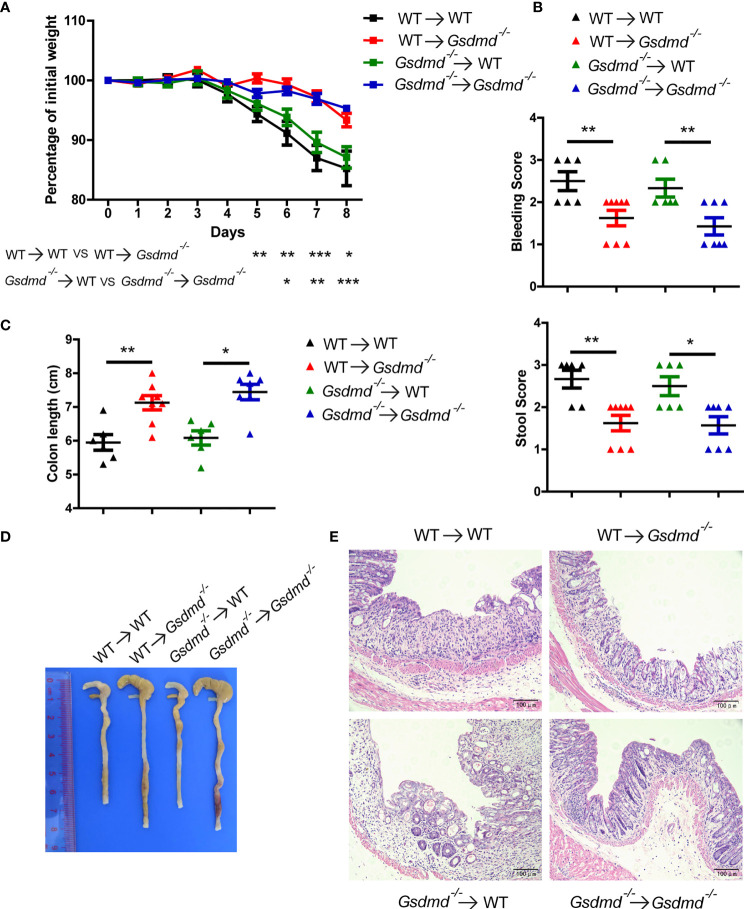
GSDMD in nonhematopoietic cells is crucial for promoting DSS-induced injury. **(A)** During the progression of DSS-induced colitis, body weight change of the following BM-transplanted mice: WT → WT (n = 6), WT → *Gsdmd^-/-^* (n = 8), *Gsdmd^-/-^* → WT (n = 6), and *Gsdmd^-/-^ → Gsdmd^-/-^* mice (n=7). **(B)** Bleeding score and stool score of the transplanted mice on Day 6 of the colitis model as in **(A)**. **(C, D)** Colon length **(C)** and the macroscopic view **(D)** of the transplanted mouse colon on Day 8 of the colitis model as in **(A)**. **(E)** H&E staining of the representative mouse colon on Day 8 of the colitis model as in **(A)** (200× magnification). Data are representative of two **(A–E)** independent experiments (mean ± SEM in **A-C**). *p < 0.05, **p < 0.01, ***p < 0.001 by Student’s t test.

To determine which cell populations are critical for GSDMD-mediated DSS-induced colitis, we generated four groups of GSDMD bone marrow chimeras. Irradiated WT recipient mice receiving WT or *Gsdmd^-/-^* bone marrow had similar body weight loss (**Figure 2A**), clinical score (**Figure 2B**), colon length (**Figures 2C, D** **)**, and histological damage (**Figure 2E**), suggesting that gut-infiltrating immune cells are not important for GSDMD-mediated colitis promotion. However, compared to the reconstituted WT recipient mice, irradiated *Gsdmd^-/-^* recipient mice receiving WT or *Gsdmd^-/-^* bone marrow showed reduced body weight loss (**Figure 2A**), clinical scores (**Figure 2B**), histological damage (**Figure 2E**), and increased colon length (**Figures 2C, D** **)**, indicating that IECs are critical for the GSDMD-mediated promotion of colitis. Collectively, these data indicate that GSDMD expression in IECs is critical for GSDMD-mediated colitis promotion.

### GSDMD Activation Is Required for GSDMD-Mediated Colitis Development

**Figure 3:**
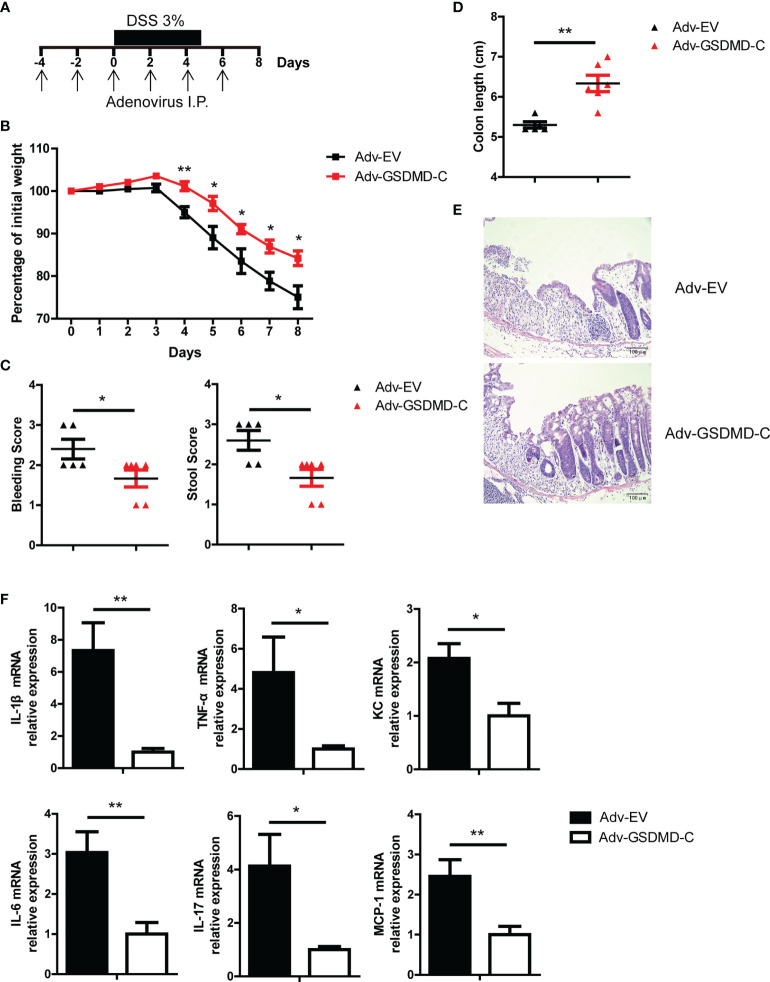
Adenovirus-mediated GSDMD-C expression protects mice from DSS-induced colitis. **(A)** Scheme of adenovirus-mediated GSDMD-C expression in mice. The mice were intraperitoneally (I.P.) injected empty control adenovirus (Adv-EV) or GSDMD-C expressing adenovirus (Adv-GSDMD-C) every other day, and 4 days later, colitis was induced in the mice with DSS solution. **(B)** Body weight change of WT mice treated with Adv-EV (n = 5) or Adv-GSDMD-C (n = 6) during the progression of DSS-induced colitis. **(C)** Bleeding score and stool score of Adv-EV- or Adv-GSDMD-C-treated WT mice on Day 6 of the colitis model as in **(B)**. **(D, E)** Colon length **(D)** and H&E staining **(E)** of Adv-EV- or Adv-GSDMD-C-treated WT mouse colons on Day 8 of the colitis model as in **(B)** (200× magnification). **(F)** Quantitative mRNA expression of inflammatory genes as indicated from the colons of Adv-EV- or Adv-GSDMD-C-treated WT mice on Day 8 of the colitis model as in **(B)**. Data are representative of three **(B–F)** independent experiments (mean ± SEM in **B–D, F**). *p < 0.05, **p < 0.01 by Student’s t test.

GSDMD-N can form membrane pores to mediate cell pyroptosis, while GSDMD-C can bind to GSDMD-N to maintain GSDMD in an unfunctional state (28, 31, 32). Moreover, Shi et al, reported that overexpression of GSDMD-C suppressed LPS-induced pyroptosis in HeLa cells due to *trans*-inhibition of endogenous GSDMD-N generated from caspase-4 cleavage (28). In our study, we found that adenovirus-mediated GSDMD-C (Adv-GSDMD-C) overexpression suppressed LPS-induced pyroptosis in HT-29 cells, an intestinal epithelial cell line (**Supplementary Figures 4A, B** **)**. To determine whether the pathological role of GSDMD in acute colitis is dependent on GSDMD activation, we used Adv-GSDMD-C overexpression in the mouse colon and then challenged the mice with DSS. The mice were intraperitoneally (i.p.) injected with empty control adenovirus (Adv-EV) or Adv-GSDMD-C every other day; 4 days later, the mice were challenged with DSS (**Figure 3A**). We found that GSDMD-C was significantly expressed in mouse colons that were infected by Adv-GSDMD-C several times (**Supplementary Figure 4C** and **Supplementary Figure 5C**). We detected the GSDMD expression and found that Adv-EV or Adv-GSDMD-C did not change GSDMD expression of mouse colon (**Supplementary Figure 4D**). The Adv-GSDMD-C treated mice had less body weight loss, and exhibited lower clinical scores, longer colon lengths and decreased colon injury than the Adv-EV treated mice (**Figures 3B-E**). The induced inflammatory genes also decreased in the Adv-GSDMD-C treated mice compared to the Adv-EV treated mice (**Figure 3F**). Next, we investigated whether Adv-GSDMD-C affects IEC pyroptosis during colitis. TUNEL staining showed that Adv-GSDMD-C largely suppressed IEC death (**Supplementary Figures 5A, B** **)**. Immunoblot analysis showed that the expression of cleaved Caspase 3 was similar in both whole colon and IECs between Adv-EV- and Adv-GSDMD-C treated mice, suggesting that Adv-GSDMD-C suppressed IEC pyroptosis during DSS-induced colitis (**Supplementary Figure 5C**). Collectively, our data show that GSDMD-C protects mice from acute colitis.

### *Gsdmd^-/-^* Mice Have Fewer Intestinal Firmicutes, but This Is Not Associated With Their Hyposensitivity Toward DSS-Induced Colitis

Various papers reported that gene-deficient mice had gut microbiota composition alterations, and some of the alterations were associated with IBD development (4, 20, 27, 43, 44). To determine whether *Gsdmd^-/-^* mice have changed gut microbiota composition, we used 16S rDNA real-time PCR to examine gut microbiota extracted from fecal samples of WT and *Gsdmd^-/-^* mice without DSS treatment. Four major intestinal bacterial phyla (Bacteroidetes, Firmicutes, Actinobacteria and Proteobacteria) and a colitis-associated bacteria phylum (TM7), as well as their representative classes, genera, or species, were checked (7, 44). Interestingly, the numbers of Actinobacteria, Bacteroidetes, Proteobacteria and TM7 were not significantly altered (**Supplementary Figure 6A**). A previous study reported that *E. coli* burden was obviously higher in inflammasome-deficient mice (27). However, we found that the *E. coli* burden was not significantly changed in *Gsdmd^-/-^* mice (**Supplementary Figure 6A**). Interestingly, the number of Firmicutes was reduced in *Gsdmd*-deficient mice (**Supplementary Figure 6A**). Segmented filamentous bacteria (SFB), Enterococcus and Lactobacillus, which belong to the Firmicutes phylum, were not altered in *Gsdmd*-deficient mice; however, other members of Firmicutes, such as Clostridium cluster IV, Clostridium cluster XIVa, *Eubacterium rectale* (EREC), and Bacillus, which are potential beneficial microbiota for colitis (9), were obviously reduced in *Gsdmd^-/-^* mice. To assess whether the altered composition of the gut microbiota is important for GSDMD-mediated colitis promotion, WT and *Gsdmd^-/-^* mice were cohoused for 4 weeks, and their gut microbiota were assessed before DSS challenge. As expected, the numbers of Firmicutes, Clostridium cluster IV, Clostridium cluster XIVa, *EREC* and Bacillus in *Gsdmd*-deficient mice equaled that of cohoused WT mice (**Supplementary Figure 6B**). In agreement with the results of separately housed mice (**Figures 1G–K**), despite similar gut microbiota compositions, cohoused *Gsdmd^-/-^* mice showed reduced body weight loss compared to WT mice during DSS-induced colitis (**Supplementary Figure 6C**). The bleeding score, and stool score decreased and colon length increased in *Gsdmd*-deficient mice (**Supplementary Figures 6D, E** **)**. Histological damage was also ameliorated in *Gsdmd^-/-^* mice (**Supplementary Figure 6F**). Together, these results indicate that although *Gsdmd*-deficient mice have fewer Firmicutes, this is not important for the reduced colitis severity in *Gsdmd*-deficient mice.

### Dysregulated Commensal *E. coli* Activates GSDMD to Promote DSS-Induced Colitis

**Figure 4:**
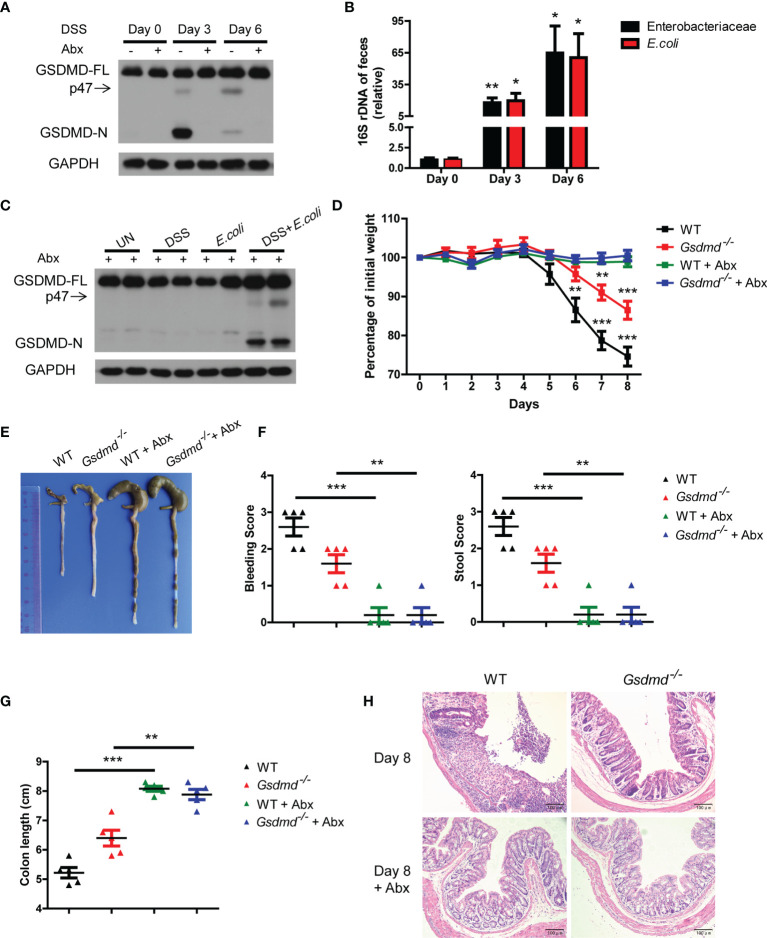
In DSS-induced colitis, the dysregulated microbiota is responsible for GSDMD activation. **(A)** Immunoblot analysis of GSDMD expression in whole colons of WT mice treated with or without a cocktail of antibiotics (Abx) at the indicated times during DSS-induced colitis. **(B)** Real-time PCR analysis of 16S rRNA genes of Enterobacteriaceae or *E. coli* species in the feces of WT mice at the indicated times in the colitis model (n = 6 or 7/group). **(C)** Immunoblot analysis of GSDMD expression from whole colons of Abx-treated WT mice, which were given with or without DSS solution or 10^10^ CFU of *E. coli*, on Day 6 during DSS-induced colitis. **(D)** Body weight change of WT or *Gsdmd^-/-^* mice treated with or without Abx during the progression of DSS-induced colitis (n = 5/group). **(E)** Macroscopic view of the representative mouse colon on Day 8 of the colitis model as in **(D)**. **(F)** Bleeding score and stool score of WT or *Gsdmd^-/-^* mice treated with or without Abx on Day 6 of the colitis model as in **(D)** (n = 5/group). **(G)** The colon length of WT or *Gsdmd^-/-^* mice treated with or without Abx on Day 8 of the colitis model as in **(D)** (n = 5/group). **(H)** H&E staining of the representative mouse colon on Day 8 of the colitis model as in **(D)** (200× magnification). Data are representative of two **(A–C)** or three **(D–H)** independent experiments (mean ± SEM in **B, D, F, G**). *p < 0.05, **p < 0.01, ***p < 0.001 by Student’s t test.

Although the different microbiota compositions of WT and *Gsdmd^-/-^* mice are not important for GSDMD-mediated colitis development, the gut microbiota is dysregulated during acute colitis (3, 8). Therefore, we asked whether the activation of GSDMD is regulated by dysregulated gut microbiota during DSS-induced colitis. We removed gut microbiota with a cocktail of antibiotics (Abx), and we found that the expression of GSDMD-FL was not altered in the colons of WT mice treated with antibiotics during DSS-induced colitis; however, cleaved GSDMD-N was almost completely blocked (**Figure 4A** and **Supplementary Figure 7A**), which indicates that the gut microbiota contributes to GSDMD activation. We and others have previously reported that Enterobacteriaceae, primarily *Escherichia coli* (*E. coli*), which belongs to Enterobacteriaceae, largely overgrew during acute colitis (7, 8, 45, 46), to determine which bacterial species are responsible for GSDMD activation, we asked whether the overgrowth *E. coli* is responsible for GSDMD activation. In agreement with previous reports, we found that the numbers of Enterobacteriaceae and *E. coli* largely increased in WT mice during colitis (**Figure 4B**). To determine whether the overgrowth of *E. coli* can activate GSDMD, we isolated *E. coli* from the feces of DSS-induced mice, and then the Abx-treated mice were given *E. coli* by gavage and rectal administrations. We found that DSS plus *E. coli* activated GSDMD in mice treated with antibiotics, whereas *E. coli* monocolonization alone did not (**Figure 4C**), which was consistent with our previous finding (7). These data suggest that the dysregulated microbiota, especially *E. coli*, is responsible for GSDMD activation during DSS-induced colitis.

Next, we investigated whether GSDMD is involved in microbiota-driven colitis development. We found that Abx-treated WT and *Gsdmd^-/-^* mice had almost no body weight loss during the DSS-induced colitis model (**Figure 4D**). Although untreated *Gsdmd^-/-^* mice lost less body weight, and exhibited longer colons and lower clinical scores than untreated WT mice, microbiota depletion completely attenuated the phenotypic differences between WT and *Gsdmd^-/-^* mice during DSS-induced colitis (**Figures 4D-H**). Consistently, microbiota removal also blocked the increased production of proinflammatory genes in both WT and *Gsdmd^-/-^* mice (**Supplementary Figure 7B**). These findings show that GSDMD is most likely critical for microbiota-mediated contribution to colitis development. Collectively, our data suggest that dysregulated commensal *E. coli* mediates GSDMD activation, which in turn promotes colitis development.

### Reduced IL-1β Is Not Important for Protection Against Colitis in *Gsdmd*-Deficient Mice

**Figure 5:**
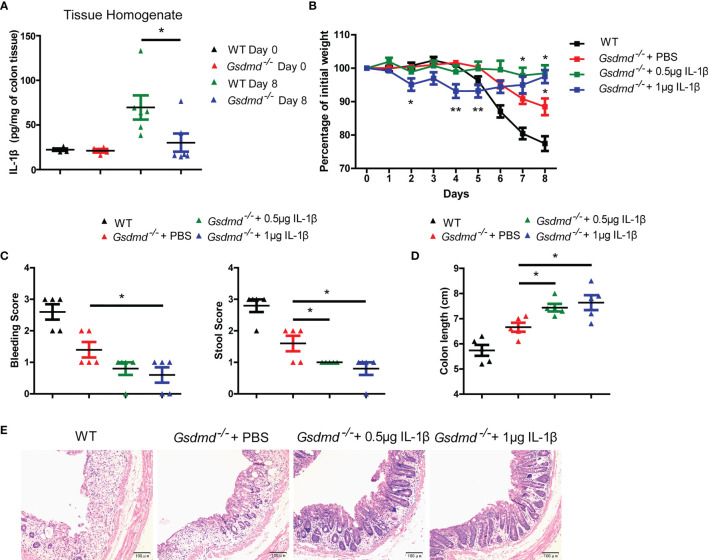
Reduced IL-1β is not responsible for the protective effect against colitis in *Gsdmd*-deficient mice. **(A)** ELISA analysis of IL-1β protein levels in colon homogenates from WT or *Gsdmd^-/-^* mice on Day 0 or Day 8 of the colitis model. **(B)** *Gsdmd^-/-^* mice simultaneously received a daily injection of 0.5 μg IL-1β, 1 μg IL-1β or PBS, while WT mice were injected with PBS (n = 5/group). The body weight change of the above mice was monitored during the progression of DSS-induced colitis. **(C)** Bleeding score and stool score of WT or *Gsdmd^-/-^* mice on Day 6 of the colitis model as in **(B)**. **(D, E)** Colon length **(D)** and H&E histology **(E)** of WT or *Gsdmd^-/-^* mouse colons on Day 8 of the colitis model as in **(B)**. Data are representative of two **(A–E)** independent experiments (mean ± SEM in **A–D**). *p < 0.05, **p < 0.01 by Student’s t test.

IL-1β is a critical proinflammatory cytokine produced by the inflammasome, and its role in colitis remains controversial (17, 24, 25). We found that the production of IL-1β was reduced in *Gsdmd^-/-^* mice compared to WT mice in DSS-induced colitis (**Figure 1M** and **Figure 5A**), so we asked whether the decreased IL-1β expression is important for the protective effect in *Gsdmd*-deficient mice. We intraperitoneally injected *Gsdmd^-/-^* mice with 0.5 μg or 1 μg recombinant mouse IL-1β every day (from Day 1 to Day 6) and challenged the mice with DSS. Surprisingly, we found that 1 μg IL-1β treated *Gsdmd^-/-^* mice showed a slightly more body weight loss from Day 2 to Day 5 than PBS-treated *Gsdmd^-/-^* mice; however, the 1 μg IL-1β-treated *Gsdmd^-/-^* mice exhibited obviously heavier body weight on Day 8 (**Figure 5B**). The 0.5 μg IL-1β-treated *Gsdmd^-/-^* mice also showed heavier body weights on Day 8 and similar body weights on Day 2, Day 4 and Day 5 compared to PBS-treated *Gsdmd^-/-^* mice (**Figure 5B**). Moreover, the IL-1β treated *Gsdmd^-/-^* mice showed lower clinical scores (**Figure 5C**), longer colon lengths (**Figure 5D**) and reduced colonic injury (**Figure 5E**). Collectively, our data indicate that exogenous IL-1β protects *Gsdmd^-/-^* mice from acute colitis, and thus the decreased IL-1β is not the major reason for the protection against colitis in *Gsdmd*-deficient mice.

### GSDMD Promotes Acute Colitis Development by Mediating IL-18 Release

**Figure 6:**
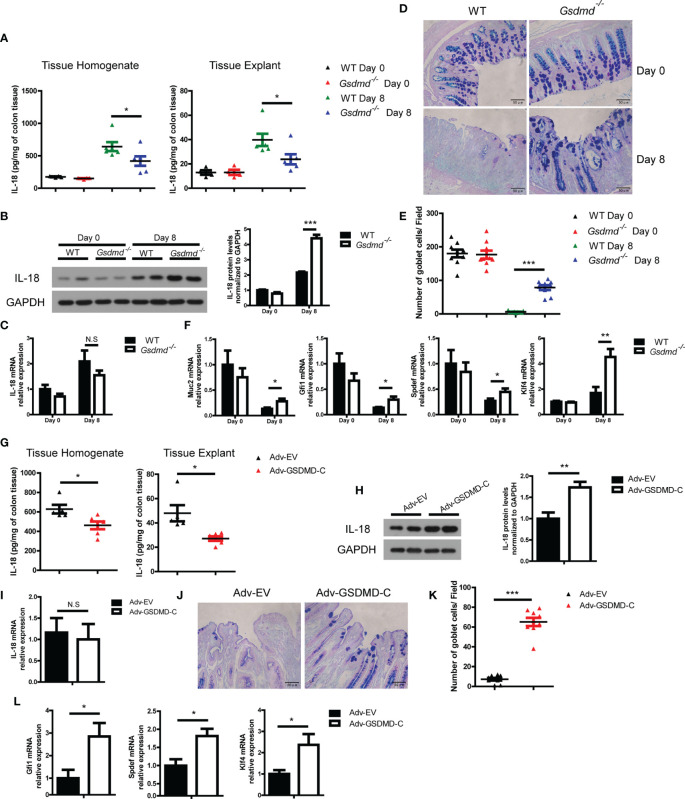
GSDMD increases IL-18 release during DSS-induced colitis. **(A)** ELISA analysis of IL-18 protein levels in WT or *Gsdmd^-/-^* mouse colon homogenates or cultured colon tissue on Day 0 or Day 8 of the colitis model, as shown in **Figure 1G** (n=4/6 per group). **(B)** Immunoblot analysis of IL-18 expression in WT or *Gsdmd^-/-^* mouse colons on Day 0 or Day 8 of the colitis model, as shown in **Figure 1G**. The quantitative analysis of IL-18 is shown on the right side. **(C)** Quantitative mRNA expression of IL-18 from WT or *Gsdmd^-/-^* mouse colons on Day 0 or Day 8 of the colitis model, as shown in **Figure 1G**. **(D, E)** AB-PAS staining of the representative colons from the mice on Day 0 or Day 8 of the colitis model, as shown in **Figure 1G** (400× magnification). The number of goblet cells per field **(E)** was determined as in **(D)** (n = 9/group). **(F)** Quantitative mRNA expression of the indicated genes from the colon of WT or *Gsdmd^-/-^* mice on Day 0 or Day 8 of the colitis model, as shown in **Figure 1G**. **(G)** ELISA analysis of IL-18 protein levels in the colons of Adv-EV- (n = 5) or Adv-GSDMD-C-treated (n = 6) WT mice on Day 8 of the colitis model, as shown in **Figure 3B**. **(H)** Immunoblot analysis of IL-18 expression in the colons of Adv-EV- or Adv-GSDMD-C-treated WT mice on Day 8 of the colitis model, as shown in **Figure 3B**. The quantitative analysis of IL-18 is shown on the right side. **(I)** Quantitative mRNA expression of IL-18 in the colons of Adv-EV- or Adv-GSDMD-C-treated WT mice on Day 8 of the colitis model, as shown in **Figure 3B**. **(J, K)** AB-PAS staining of the representative colons from the Adv-EV- or Adv-GSDMD-C-treated WT mouse colon on Day 8 of the colitis model, as shown in **Figure 3B** (400× magnification). The number of goblet cells per field **(K)** was determined as in **(J)** (n = 9/group). **(L)** Quantitative mRNA expression of the indicated genes from the colons of Adv-EV- or Adv-GSDMD-C-treated WT mice on Day 8 of the colitis model, as shown in **Figure 3B**. Data are representative of two **(A, B, G, H)** or three **(C-F, I–L)** independent experiments (mean ± SEM in **A–C, E–I, K–L**). *p < 0.05, **p < 0.01, ***p < 0.001 by Student’s t test. N.S, no significance.

IL-18 promotes DSS-induced colitis by driving goblet cell loss (19), and Miao et al, reported that GSDMD was responsible for IL-18 release in tubular epithelial cells during acute kidney injury (47). We asked whether GSDMD promotes IL-18 release during colitis. We found that the concentration of IL-18 in colon tissue homogenate or culture colon explant supernatant was significantly reduced in *Gsdmd*-deficient mice during colitis (**Figure 6A**). To determine whether the reduced IL-18 protein level is due to reduced IL-18 maturation or reduced IL-18 release, we checked intracellular mature IL-18 expression by immunoblots and found that the intracellular mature IL-18 level in *Gsdmd*-deficient mouse colons was greater than that in WT mouse colons, suggesting that the accumulation of intracellular mature IL-18 increases in *Gsdmd*-deficient colons (**Figure 6B**). Moreover, the mRNA level of IL-18 was not altered between WT and *Gsdmd^-/-^* mice (**Figure 6C**). These data suggest that the suppressed IL-18 release from the cytoplasm of *Gsdmd^-/-^* mouse colon most likely accounts for the reduced IL-18 level in colon tissue homogenate or culture medium, and GSDMD might not affect the transcription or maturation of IL-18 during colitis. Next, we checked whether GSDMD affects goblet cell damage during DSS-induced colitis. As expected, compared to WT mice, the number of goblet cells in *Gsdmd*-deficient mice was better preserved during DSS-induced colitis (**Figures 6D, E** **)**. During DSS-induced colitis, the mRNA level of Muc2, a mucin protein that was primarily produced by goblet cells, was higher in *Gsdmd*-deficient mice than in WT mice (**Figure 6F**). IL-18 drives goblet cell loss by preventing goblet cell maturation (19). In our setting, we indeed found that the mRNA levels of goblet cell maturation-related transcription factors Gfi1, Spdef, and Klf4 were higher in *Gsdmd*-deficient mice than in WT mice during colitis (**Figure 6F**). To determine whether GSDMD activation is required for IL-18 release, we assessed IL-18 release in Adv-GSDMD-C treated mice. We found that the protein level of IL-18 from tissue homogenate or culture colon explant medium was decreased in GSDMD-C treated mice (**Figure 6G**). The intracellular mature IL-18 protein level was increased in the colon of Adv-GSDMD-C treated mice (**Figure 6H**). Meanwhile, the mRNA level of IL-18 was not altered (**Figure 6I**). These data suggest that GSDMD-C overexpression suppresses IL-18 release during DSS-induced colitis. Overexpression of GSDMD-C also increased the number of goblet cells in mice during colitis (**Figures 6J, K** **)**. The mRNA levels of Gfi1, Spdef and Klf4 also increased in Adv-GSDMD-C treated mice (**Figure 6L**). Collectively, these data suggest that GSDMD promotes IL-18 release during colitis; and that the activation of GSDMD is required for its release.

**Figure 7:**
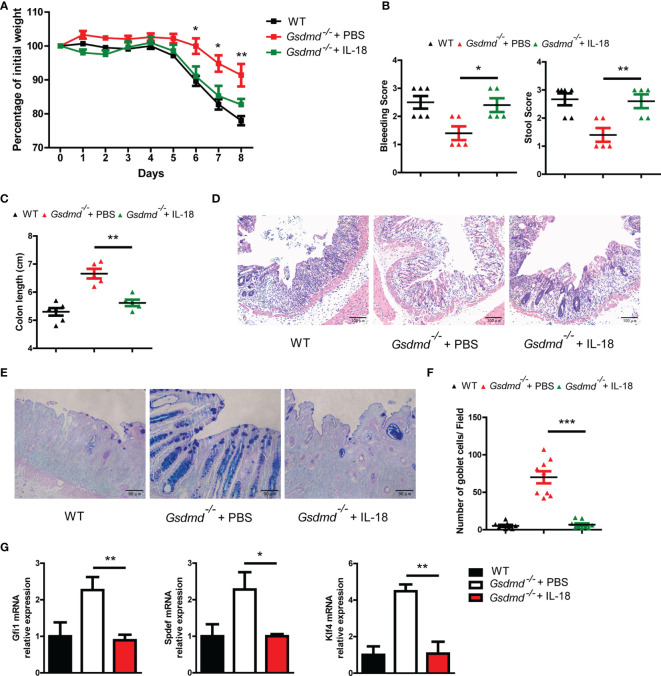
IL-18 is involved in GSDMD-mediated colitis promotion. **(A)** *Gsdmd^-/-^* mice simultaneously received a daily injection of 1 μg of IL-18 or PBS, while WT mice were injected with PBS (n = 5/6 per group). The body weight change of the above mice was monitored during the progression of DSS-induced colitis. **(B)** Bleeding score and stool score of the indicated mice on Day 6 of the colitis model as in **(A)**. **(C)** Colon length of the indicated mice on Day 8 of the colitis model as in **(A)**. **(D)** H&E histology of representative mouse colons on Day 8 of the colitis model as in **(A)** (200× magnification). **(E, F)** AB-PAS staining of the representative colons from the mice on Day 8 of the colitis model as in **(A)** (400× magnification). The number of goblet cells per field **(F)** was determined as in **(E)** (n = 9/group). **(G)** Quantitative mRNA expression of the indicated genes from the mouse colon on Day 8 of the colitis model as in **(A)**. Data are representative of two **(A–G)** independent experiments (mean ± SEM in **A–C, F–G**). *p < 0.05, **p < 0.01, ***p < 0.001 by Student’s t test.

To determine whether GSDMD exacerbates colitis pathology by inducing IL-18 release, we injected intraperitoneally *Gsdmd*-deficient mice at a concentration of 1 μg recombinant mouse IL-18 per mouse daily during DSS colitis. We found that exogenous IL-18 obviously decreased *Gsdmd^-/-^* mouse body weight, and the reduced body weight was similar to that of WT mice (**Figure 7A**). The IL-18 treated *Gsdmd^-/-^* mice showed a higher clinical score (**Figure 7B**), shorter colon length (**Figure 7C**), and increased colonic injury (**Figure 7D**). Moreover, exogenous IL-18 reduced the goblet cell number (**Figures 7E, F** **)**, and decreased the mRNA levels of Gfi1, Spdef, and Klf4 in *Gsdmd* deficient mice (**Figure 7G**). These data show that exogenous IL-18 promotes colitis severity in *Gsdmd*-deficient mice. Together, our data suggest that GSDMD promotes IL-18 release during colitis; and that the released IL-18 mediates colitis development by promoting goblet cell loss.

## Discussion

The interaction between the host and gut microbiota is critical for maintaining gut homeostasis (4, 48). When the homeostatic balance is compromised, the gut microbiota will be dysregulated and trigger the development of various inflammatory diseases, including colitis (7, 8, 49). However, the molecular mechanism for microbiota-mediated regulation of colitis development is largely unknown. Here, we indicated that dysregulated microbiota-driven GSDMD activation promoted colitis development by inducing IL-18 release.

The roles of the inflammasome in colitis are controversial. *Nlrp3*- and *Caspase 1*-deficient mice were reported to show ameliorated colitis severity (14, 15, 21), while others reported that DSS-induced colitis was aggravated in these mice (16, 22, 23). Similarly, the roles of ASC, Caspase 11, IL-18, and IL-1β in colitis remain debatable (17–21, 24, 25). GSDMD is another critical inflammasome protein that mediates cell pyroptosis (28, 29). Here, our data found that GSDMD deficiency protected mice from DSS-induced colitis, which is consistent with a very recent report (50). Interestingly, Ma et al. found that DSS-induced colitis was aggravated in *Gsdmd*-deficient mice (51). The possible reasons for the seemingly controversial data as follows: i) We used different strategies to induce colitis. Mice were treated with 3% DSS for 5 days in our settings while 2.5% DSS challenged for 6 days was used in their study. ii) The differences in mouse housing and feeding among animal facilities probably led to different gut microbiota compositions and then caused different colitis severities.

IECs are the first lie of defense against pathogens and pathobionts, and IEC damage is the critical factor promoting colitis development (52). In the present study, we found that GSDMD was highly expressed in IECs but not LPLs. To investigate which cell populations are critical for GSDMD-mediated colitis promotion, we used a bone-barrow transfer assay to generate GSDMD bone-barrow chimeras. Our data showed that GSDMD in IECs was responsible for GSDMD-mediated colitis promotion, while gut infiltrating immune cell-derived GSDMD was not involved in this process. Our findings further confirm that IECs play critical roles in inflammasome-mediated colitis.

In our study, we found that GSDMD was activated in DSS-induced colitis. To investigate whether the activation of GSDMD is required for GSDMD-mediated colitis development, we used Adv-mediated GSDMD-C expression, which suppressed GSDMD-mediated cell pyroptosis by binding GSDMD-N, in WT mouse colons. We found that Adv-GSDMD-C obviously suppressed colitis development, suggesting that GSDMD activation is required for GSDMD-mediated colitis development. Furthermore, we investigated how GSDMD is activated in the mouse intestinal tract. Previously, we and others found that gram-negative bacteria Enterobacteriaceae, particularly *E. coli* species, largely overgrew during DSS-induced colitis and then exacerbated colitis pathology (7, 8, 45, 46). Here we found that Enterobacteriaceae and *E. coli* largely increased during DSS-induced colitis. When the gut microbiota, including *E. coli*, was removed by antibiotics, GSDMD activation was completely inhibited in the colon of DSS-treated mice. When the antibiotic-treated mice were transplanted with *E. coli* and challenged with DSS, the DSS plus *E. coli* group showed obvious GSDMD activation, while DSS or *E. coli* alone could not activate GSDMD. The data suggest that the *E. coli* translocates to the basolateral surface of IECs to directly induce GSDMD activation in IECs after DSS-mediated epithelium damage, while *E. coli* cannot activate GSDMD in IECs without DSS-induced epithelium damage (11). Collectively, these data suggested that the microbiota was dysregulated during colitis; and that the dysregulated microbiota, particularly *E. coli*, mediated colitis development by activating GSDMD.

IL-1β is an important proinflammatory cytokine produced by the inflammasome. Some reports found that IL-1β enhanced colitis severity in animal models (24, 25), while others reported that *Il1β* ^-/-^ and *Il1r1* ^-/-^, the IL-1α and IL-1β receptor, mice displayed exacerbated severity in DSS-induced colitis (17, 53). We found that IL-1β production was obviously reduced in *Gsdmd^-/-^* mice during DSS-induced colitis, and we asked whether the reduced pathological severity of *Gsdmd^-/-^* mice was due to reduced IL-1β secretion. We injected *Gsdmd^-/-^* mice with recombinant IL-1β and challenged these mice with DSS and found that recombinant IL-1β did not increase the severity of colitis but ameliorated the disease in *Gsdmd^-/-^* mice. The possible reason is that IL-1β is involved in repairing IECs and reconstituting epithelial barriers during colitis, which is consistent with previous report (17). Interestingly, we found that 1 μg IL-1β-treated *Gsdmd^-/-^* mice showed more body weight loss on Day 2, Day 4 and Day 5 than the control group. The possible reason is that the IL-1β-mediated intense inflammatory response leads to body weight loss, which is similar to TNF- or LPS-induced shock. Our data suggest that decreased IL-1β is not important for GSDMD-mediated colitis promotion.

The role of IL-18 in colitis is complicated. Some papers reported that IL-18 had a protective role in colitis (16, 23, 54), and some other reports showed that IL-18 had a pro-colitogenic role in experimental colitis (55–57). Nowarski et al., reported that IL-18 suppressed goblet cell maturation and then promoted colitis development (19). In our study, we found that the protein level of IL-18 was reduced in the colon culture medium of *Gsdmd^-/-^* mice during DSS-induced colitis. Furthermore, we found that the reduced IL-18 in the colon culture medium was due to reduced IL-18 release but not reduced mRNA levels or decreased IL-18 maturation. Exogenous IL-18 increased colonic injury in *Gsdmd^-/-^* mice during DSS-induced colitis. In addition, IL-18 reduced the goblet cell number in *Gsdmd^-/-^* mice. These data indicate that GSDMD promotes colitis development by enhancing IL-18 release.

How does GSDMD promote IL-18 release? Miao et al., reported that Caspase 11 cleaved GSDMD into GSDMD-N during acute kidney injury, and then the cleaved GSDMD-N translocated onto plasma membrane to form membrane pores, which triggered cell proptosis and IL-18 release from primary cultured renal tubular epithelial cells (47). We hypothesized that GSDMD might play a similar role in acute kidney injury and acute colitis through a similar mechanism. Release of IL-18 was detected from both intestinal epithelial cells and lamina propria lymphocytes during colitis (19). Based on our data, we speculate that *E. coli-*derived LPS activated Caspase 11, which in turn cleaved GSDMD to GSDMD-N, forming membrane pores to release IL-18 from intestinal epithelial cells. More experiments will be performed to support the speculation in the near future.

In summary, we identified GSDMD as a critical pro-colitogenic gene. GSDMD is activated by the dysregulated microbiota and in turn mediates microbiota-driven colitis by promoting IL-18 release. Our study provides a novel mechanism for microbiota-mediated colitis development by activating GSDMD and suggests that GSDMD is a promising target for IBD therapy.

## Data Availability Statement

The original contributions presented in the study are included in the article/**Supplementary Material**. Further inquiries can be directed to the corresponding authors.

## Ethics Statement

The animal study was reviewed and approved by Institutional Biomedical Research Ethics Committee of the Guangdong Medical University.

## Author Contributions

HG, MC, PC, and YY designed the experiments. HG and PC wrote the manuscript. HG, MC, and PC conducted the experiments and analyzed the data. WH and HS helped with experiments. YZ, CZ, and JT provided reagents and technical support. HG and SL supervised the study. All authors contributed to the article and approved the submitted version.

## Funding

This work was supported by grants from the National Natural Science Foundation of China, Grant/Award Number: 81902021, 81902558, and 81801474, Guangdong Basic and Applied Basic Research Foundation, Grant/Award Number: 2019A1515011009, 2021A1515010683, 2020A1515010225, and 2021A1515010955, Shenzhen Foundation of Science and Technology, Grant/Award Number: JCYJ20180306172449376, JCYJ20180306172459580, and JCYJ20180306172502097, Shenzhen Longhua District Foundation of Science and Technology, Grant/Award Number: 201803, 2017006 and SZLHQJCYJ202002.

## Conflict of Interest

The authors declare that the research was conducted in the absence of any commercial or financial relationships that could be construed as a potential conflict of interest.

## Publisher’s Note

All claims expressed in this article are solely those of the authors and do not necessarily represent those of their affiliated organizations, or those of the publisher, the editors and the reviewers. Any product that may be evaluated in this article, or claim that may be made by its manufacturer, is not guaranteed or endorsed by the publisher.
