# Supplementary material for: Dysregulated Microbiota-Driven Gasdermin D Activation Promotes Colitis Development by Mediating IL-18 Release
Source: Front Immunol. 2021 Oct 14;12:750841. doi: 10.3389/fimmu.2021.750841 (PMC8551709; doi:10.3389/fimmu.2021.750841)
Supplement: Supplementary file 1 [file DataSheet_1.docx]

**Supplementary Table 1: Primer sequences of mouse genes for real-time PCR**

| **Gene name** | **Primer sequences (5’-3’)** |
| --- | --- |
| IL-1β | GAAATGCCACCTTTTGACAGTG |
|  | TGGATGCTCTCATCAGGACAG |
| TNF-α | CAGGCGGTGCCTATGTCTC |
|  | CGATCACCCCGAAGTTCAGTAG |
| IL-6 | CTGCAAGAGACTTCCATCCAG |
|  | AGTGGTATAGACAGGTCTGTTGG |
| IL-17 | TCAGCGTGTCCAAACACTGAG |
|  | CGCCAAGGGAGTTAAAGACTT |
| IL-18 | GTGAACCCCAGACCAGACTG |
|  | CCTGGAACACGTTTCTGAAAGA |
| KC | ACTGCACCCAAACCGAAGTC |
|  | TGGGGACACCTTTTAGCATCTT |
| MCP-1 | TAAAAACCTGGATCGGAACCAAA |
|  | GCATTAGCTTCAGATTTACGGGT |
| Caspase 1 | ACAAGGCACGGGACCTATG |
|  | TCCCAGTCAGTCCTGGAAATG |
| Caspase 11 | AGCGTTGGGTTTTTGTAGATGC |
|  | CCTTGTGAACTCTTCAGGGGA |
| BD1 | TGGAGCGGAGACAGAATCCT |
|  | AGTCTTGGACGAAGAACAGATCAA |
| BD2 | TGACCACTGCCACACCAATG |
|  | CCTGGCAGAAGGAGGACAAA |
| BD3 | TCGGTGCATTGGCAACACT |
|  | TGCAGCATTTGAGGAAAGGA |
| Reg3β | ATGGCTCCTACTGCTATGCC |
|  | GTGTCCTCCAGGCCTCTT |
| Reg3γ | CAAGGTGAAGTTGCCAAGAA |
|  | CCTCTGTTGGGTTCATAGCC |
| S100A8 | AAATCACCATGCCCTCTACAAG |
|  | CCCACTTTTATCACCATCGCAA |
| S100A9 | GCACAGTTGGCAACCTTTATG |
|  | TGATTGTCCTGGTTTGTGTCC |
| Muc2 | GCTGACGAGTGGTTGGTGAATG |
|  | GATGAGGTGGCAGACAGGAGAC |
| Gfi1 | AGAAGGCGCACAGCTATCAC |
|  | GGCTCCATTTTCGACTCGC |
| Spdef | GAATGGAGTCCTAGTCCACCC |
|  | AGCTGCTATCGTCGGGATACA |
| Klf4 | GGCGAGTCTGACATGGCTG |
|  | GCTGGACGCAGTGTCTTCTC |
| GSDMD-FL | ATGCCATCGGCCTTTGAGAAA |
|  | AGGCTGTCCACCGGAATGA |
| GSDMD-C | attgaggcggcagacttcc |
|  | tgccctagtgaggcctctaa |
| β-actin | GTGACGTTGACATCCGTAAAGA |
|  | GCCGGACTCATCGTACTCC |

**Supplementary Table 2: Specific 16S rDNA** **primer sequences for real-time PCR**

| **Bacteria** | **Primer sequences (5’-3’)** | **Reference** |
| --- | --- | --- |
| Actinobacteria | CGCGGCCTATCAGCTTGTTG | [^1^](#_ENREF_1) |
|  | ATTACCGCGGCTGCTGG |  |
| Bifidobacterium | CGGGTGAGTAATGCGTGACC | [^1^](#_ENREF_1) |
|  | TGATAGGACGCGACCCCA |  |
| Bacteroidetes | CATGTGGTTTAATTCGATGAT | [^1^](#_ENREF_1) |
|  | AGCTGACGACAACCATGCAG |  |
| Bacteroides | GGTTCTGAGAGGAGGTCCC | [^1^](#_ENREF_1) |
|  | GCTGCCTCCCGTAGGAGT |  |
| Bacteroides thetaiotaomicron | GGCAGCATTTCAGTTTGCTTG | [^1^](#_ENREF_1) |
|  | GGTACATACAAAATTCCACACGT |  |
| MIB | CCAGCAGCCGCGGTAATA | [^2^](#_ENREF_2) |
|  | CGCATTCCGCATACTTCTC |  |
| Prevotellaceae | CCAGCCAAGTAGCGTGCA | [^2^](#_ENREF_2) |
|  | TGGACCTTCCGTATTACC |  |
| Prevotella | CACRGTAAACGATGGATGCC | [^1^](#_ENREF_1) |
|  | GGTCGGGTTGCAGACC |  |
| Firmicutes | GCTGCTAATACCGCATGATATGTC | [^1^](#_ENREF_1) |
|  | CAGACGCGAGTCCATCTCAGA |  |
| Clostridial cluster IV | GCACAAGCAGTGGAGT | [^2^](#_ENREF_2) |
|  | CTTCCTCCGTTTTGTCAA |  |
| Clostridial cluster XIVa | AAATGACGGTACCTGACTAA | [^2^](#_ENREF_2) |
|  | CTTTGAGTTTCATTCTTGCGAA |  |
| EREC | ACTCCTACGGGAGGCAGC | [^2^](#_ENREF_2) |
|  | GCTTCTTAGTCAGGTACCGTCA |  |
| Bacillus | GCGGCGTGCCTAATACATGC | [^3^](#_ENREF_3) |
|  | CTTCATCACTCACGCGGCGT |  |
| SFB | AGGAGGAGTCTGCGGCACATTAGC | [^2^](#_ENREF_2) |
|  | CGCATCCTTTACGCCCAGTTATTC |  |
| Enterococcus | CCCTTATTGTTAGTTGCCATCATT | [^2^](#_ENREF_2) |
|  | ACTCGTTGTACTTCCCATTGT |  |
| Lactobacillus | AGCAGTAGGGAATCTTCCA | [^1^](#_ENREF_1) |
|  | CACCGCTACACATGGAG |  |
| Proteobacteria | CATGACGTTACCCGCAGAAGAAG | [^1^](#_ENREF_1) |
|  | CTCTACGAGACTCAAGCTTGC |  |
| Alphaproteobacteria | ACTCCTACGGGAGGCAGCAG | [^1^](#_ENREF_1) |
|  | TCTACGRATTTCACCYCTAC |  |
| Betaproteobacteria | ACTCCTACGGGAGGCAGCAG | [^1^](#_ENREF_1) |
|  | TCACTGCTACACGYG |  |
| Gammaproteobacteria | CMATGCCGCGTGTGTGAA | [^1^](#_ENREF_1) |
|  | ACTCCCCAGGCGGTCDACTTA |  |
| Epslionproteobacte-ria | TGGCGSACGGGTGAGTAATRTATAG | [^1^](#_ENREF_1) |
|  | GGAGTTTACRCWCCGAAAWGYGTC |  |
| Enterobacteriaceae | GTGCCAGCMGCCGCGGTAA | [^1^](#_ENREF_1) |
|  | GCCTCAAGGGCACAACCTCCAAG |  |
| E.coli | CATGCCGCGTGTATGAAGAA | [^1^](#_ENREF_1) |
|  | CGGGTAACGTCAATGAGCAAA |  |
| TM7 | GCAACTCTTTACGCCCAGT | [^1^](#_ENREF_1) |
|  | GAGAGGATGATCAGCCAG |  |
| Universal bacterial | ACTCCTACGGGAGGCAGCAGT | [^1^](#_ENREF_1)  [^1^](#_ENREF_1) |
|  | ATTACCGCGGCTGCTGGC |  |

**Supplementary Figure 1**


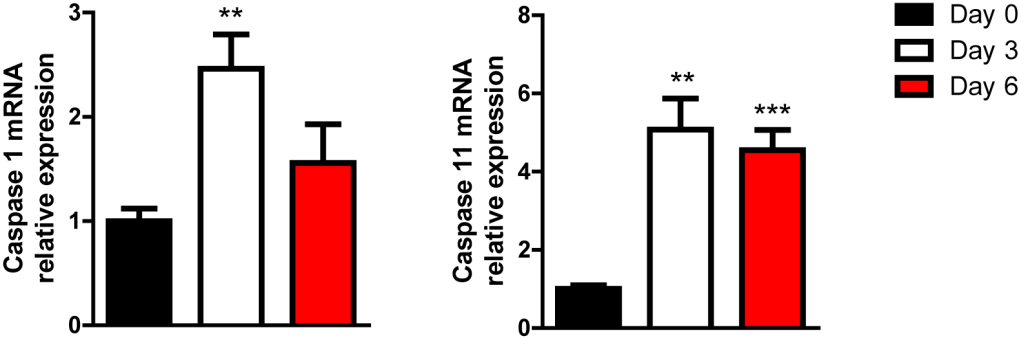


**Supplementary Figure 1: The mRNA level of Caspase 1 or Caspase 11 was induced in DSS-induced colitis**.

Quantitative mRNA expression of Caspase 1 or Caspase 11 from wild-type C57/BL6 mice colon of Day 0 (n=4), Day 3(n=6), or Day 6 (n=6) during DSS-induced colitis.

Data are representative of four independent experiments (mean±SEM). ***p* < 0.01, ****p* < 0.001 by Student’s t test.

**Supplementary Figure 2**

**
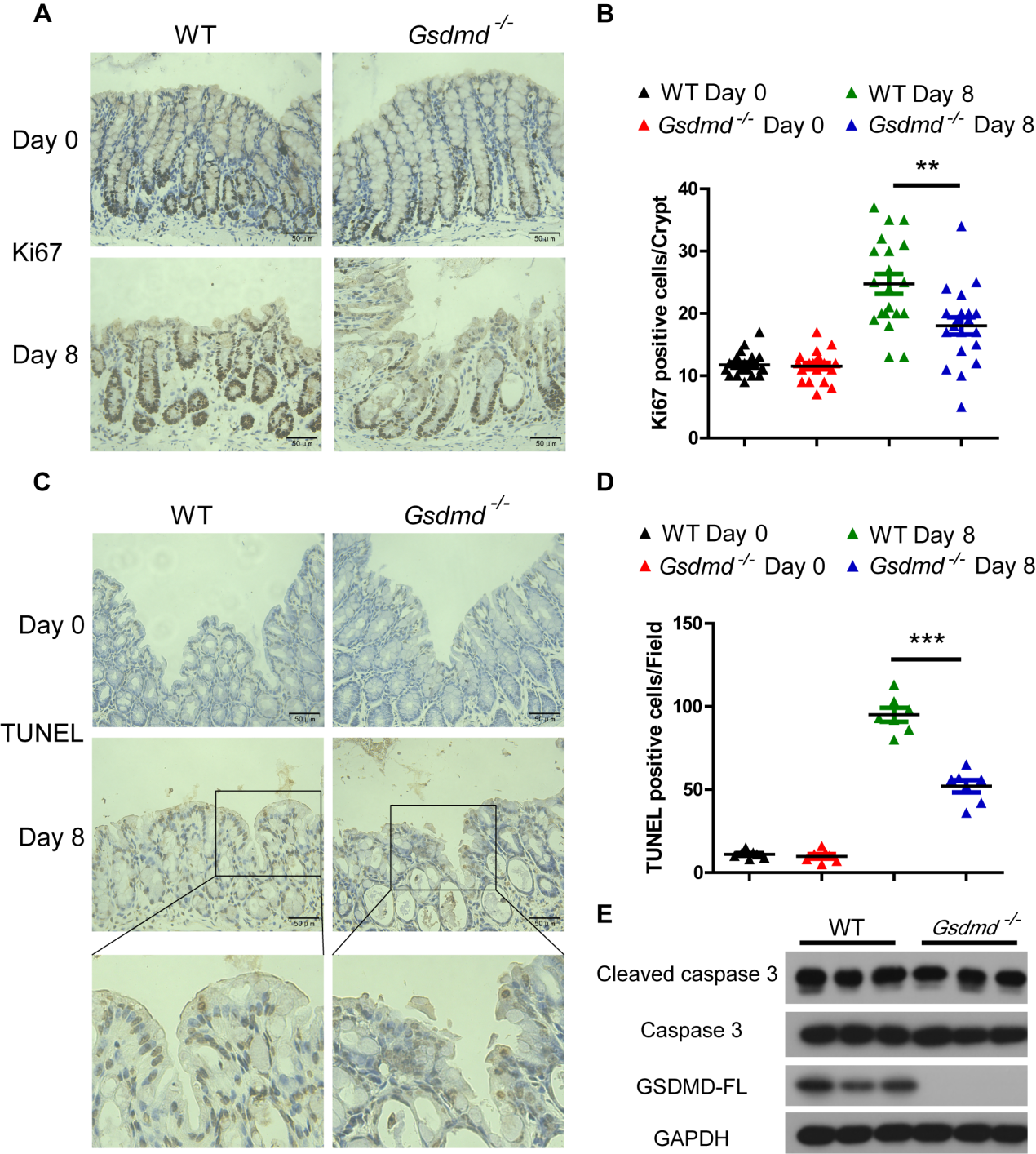
**

**Supplementary Figure 2: Intestinal epithelial cells proliferation and pyroptosis were attenuated in *Gsdmd*-deficient mice.**

(A-B) Ki-67 (A) staining of the representative colons from the WT or *Gsdmd^-/-^* mice on Day 0 or Day 8 of the colitis model (400× magnification). The number of proliferating cells per crypt (B) was determined as in (A) (n=5/group, 4 crypts were counted per mice).

(C-D) TUNEL (C) staining of the representative colons from the WT or *Gsdmd^-/-^* mice on Day 0 or Day 8 of the colitis model (400× magnification). The number of TUNEL positive cells per field (D) was determined as in (C) (n=7/group).

(E) Immunoblot analysis of Cleaved caspase 3, Caspase 3 or GSDMD-FL expression from the WT or *Gsdmd^-/-^* mice on Day 8 of the colitis model.

Data are representative of three (A-E) independent experiments (mean±SEM in B and D). ***p* < 0.01, ****p* < 0.001 by Student’s t test.

**Supplementary Figure 3**

**
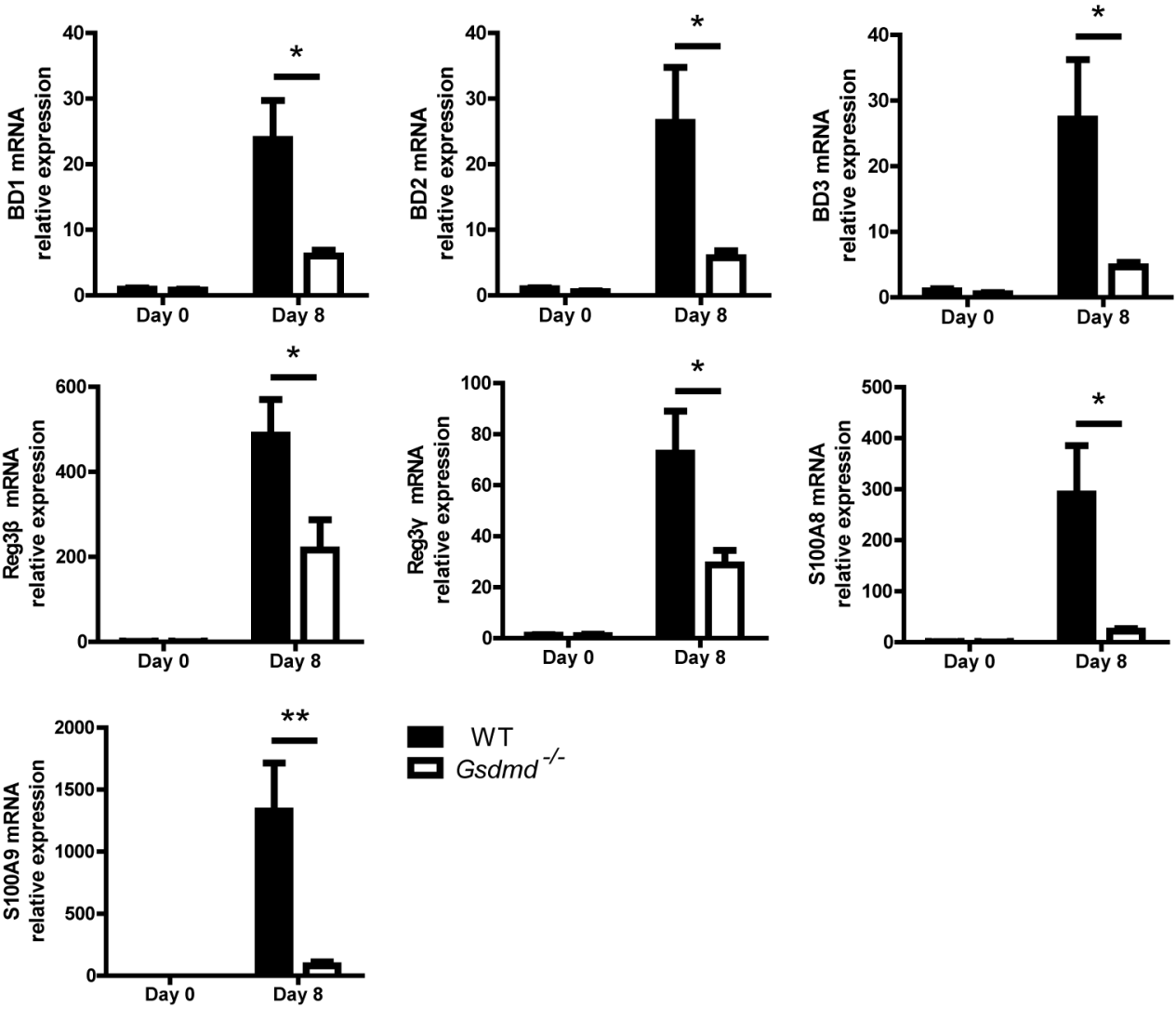
**

**Supplementary Figure 3: The antimicrobial peptides production was not the major reason for GSDMD-mediated colitis promotion.**

Quantitative mRNA expression of antimicrobial peptides (AMPs) as indicated from the WT or *Gsdmd^-/-^* mice colon on Day 0 or Day 8 of the colitis model.

Data are representative of three independent experiments (mean±SEM). **p* < 0.05, ***p* < 0.01 by Student’s t test.

**Supplementary Figure 4**

**
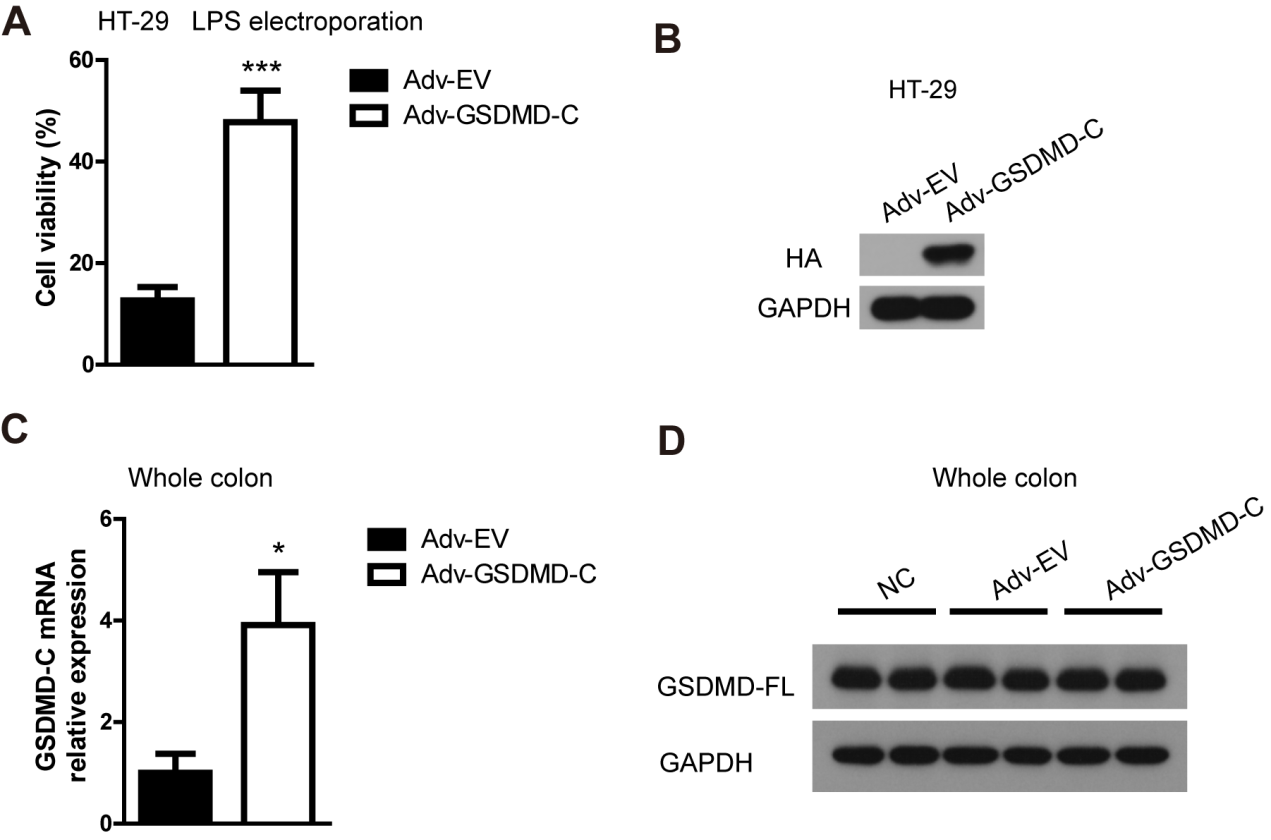
**

**Supplementary Figure 4: Adenovirus mediated-GSDMD-C expression suppressed LPS-induced pyroptosis in HT-29 cells.**

(A) Cell viability was analyzed in LPS transfected HT-29 cells infected with Adv-EV or Adv-GSDMD-C.

(B) Immunoblot analysis of HA tagged GSDMD-C expression from the HT-29 cells infected with Adv-EV or Adv-GSDMD-C.

(C) Quantitative mRNA expression of GSDMD-C on Day 8 from whole colons of WT mice treated with Adv-EV or Adv-GSDMD-C during the progress of DSS-induced colitis.

(D) Immunoblot analysis of GSDMD expression from the whole colon of the Adv-EV or Adv-GSDMD-C treated WT mice or WT mice as negative control (NC) on Day 0 of the colitis model.

Data are representative of three (A-D) independent experiments (mean±SEM in A and C). **p* < 0.05, ****p* < 0.001 by Student’s t test.

**Supplementary Figure 5**

**
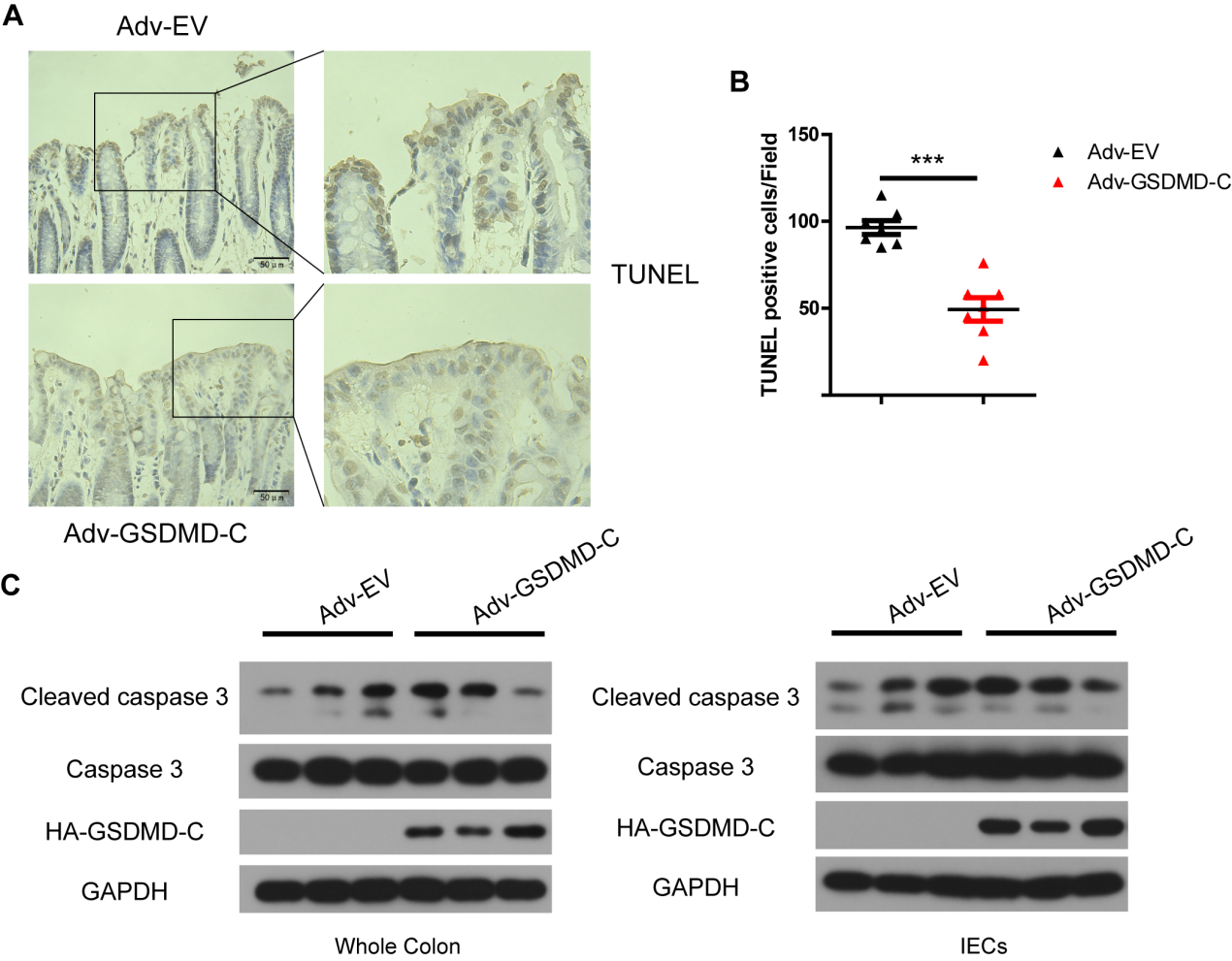
**

**Supplementary Figure 5: Adenovirus mediated-GSDMD-C expression suppressed intestinal epithelial cells pyroptosis during DSS-induced colitis.**

(A) TUNEL staining of the representative colons from the Adv-EV or Adv-GSDMD-C treated WT mice on Day 8 of the colitis model (400×magnification).

(B) The number of TUNEL positive cells per field was determined as in (A) (n=7/group).

(C) Immunoblot analysis of Cleaved caspase 3 or Caspase 3 expression from the Adv-EV or Adv-GSDMD-C treated WT mice whole colon or IECs on Day 8 of the colitis model.

Data are representative of three (A-C) independent experiments (mean±SEM in B). **p* < 0.05, ***p* < 0.01, ****p* < 0.001 by Student’s t test.

**
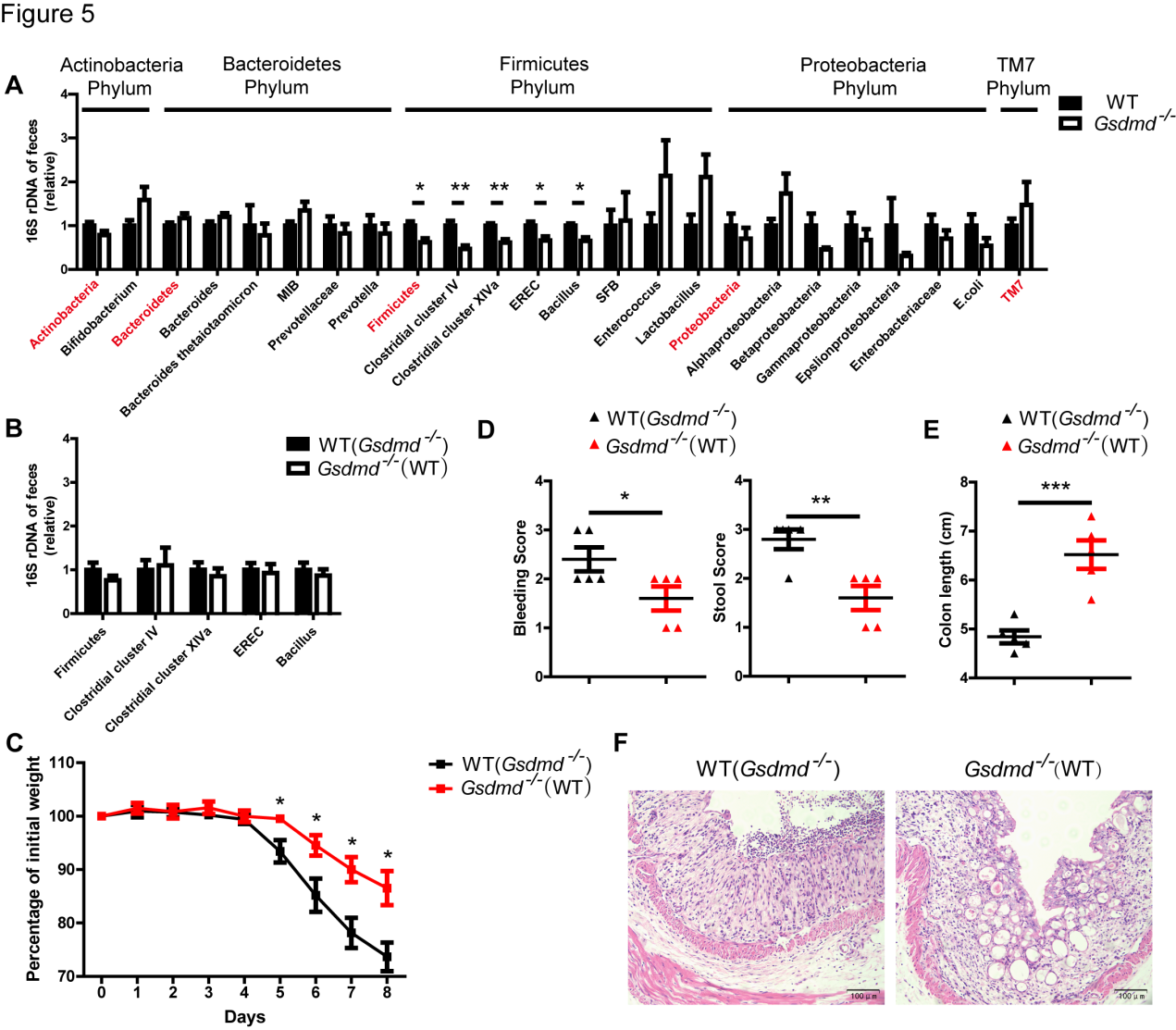
Supplementary Figure 6**

**Supplementary Figure 6: *Gsdmd-*deficient mice have less Firmicutes, but this is not responsible for the reduced colitis severity in these mice.**

(A) Real-time PCR analysis of bacterial 16S rRNA gene sequences as indicated of the fecal from WT (n=6) or *Gsdmd^-/-^* (n=7) mice.

(B) Real-time PCR analysis of bacterial 16S rRNA gene sequences as indicated of the fecal from WT (n=6) or *Gsdmd^-/-^* (n=6) mice that were cohoused for 4 weeks.

(C) During the progress of DSS-induced colitis, body weight change of the WT (n=5) or *Gsdmd^-/-^* (n=5) mice that were cohoused for 4 weeks.

(D) Bleeding score and stool score of the cohoused mice on Day 6 of the colitis model as in (C).

(E) Colon length of the cohoused mice colon on Day 8 of the colitis model as in (C).

(F) H&E staining of the representative mice colon on Day 8 of the colitis model as in (C) (200× magnification).

Data are representative of three (B-F) or four (A) independent experiments (mean±SEM in A-E). **p* < 0.05, ***p* < 0.01, ****p* < 0.001 by Student’s t test.

**Supplementary Figure 7**

**
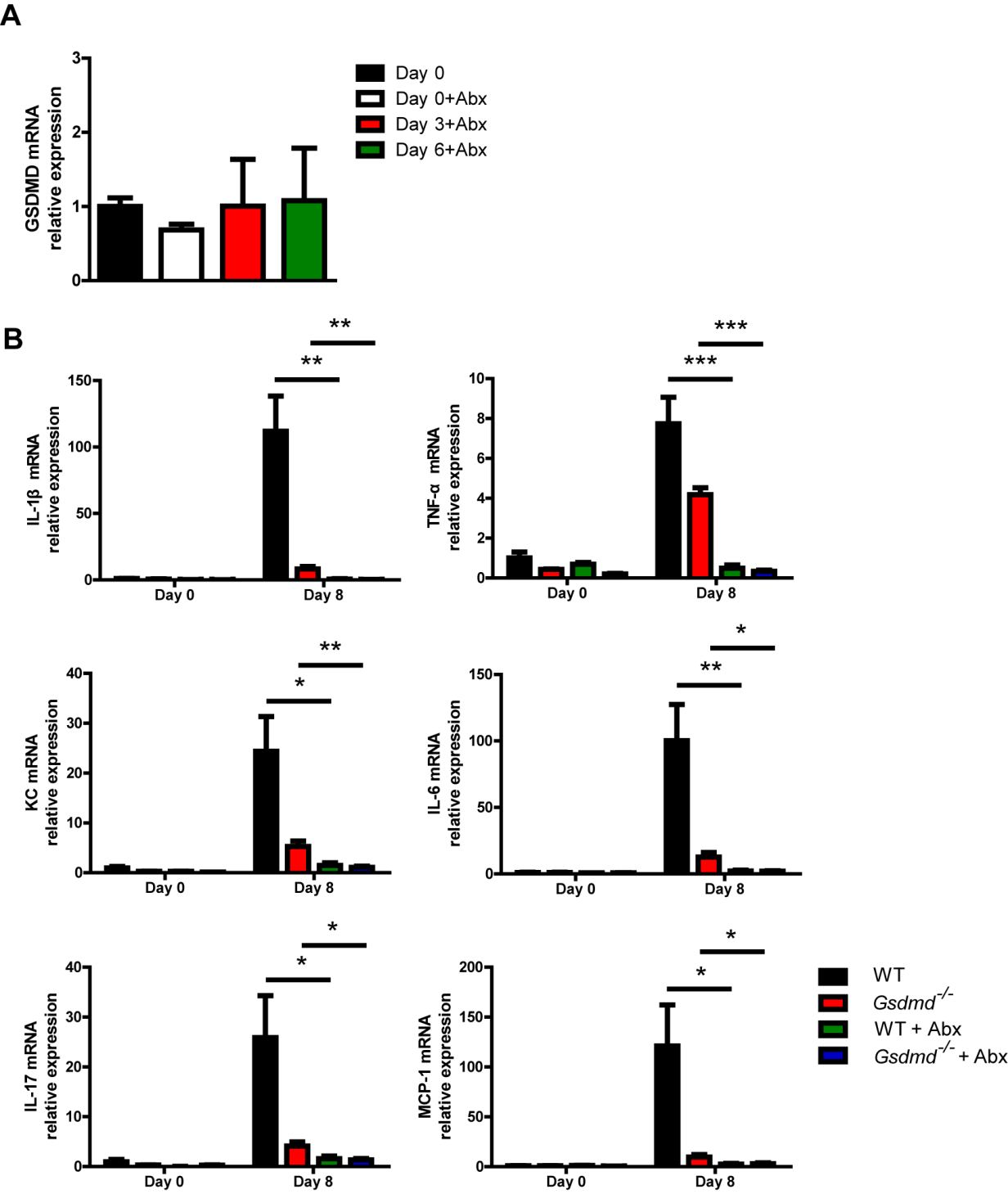
**

**Supplementary Figure 7: The increased production of pro-inflammatory genes was blocked in both wild-type and *Gsdmd^-/-^* mice treated with a cocktail of antibiotics.**

(A) Quantitative mRNA expression of GSDMD at indicated time from whole colons of wild-type C57/BL6 mice treated with or without a cocktail of antibiotics during DSS-induced colitis (n=4/group).

(B) Quantitative mRNA expression of inflammatory genes as indicated of WT or *Gsdmd^-/-^* mice treated with or without a cocktail of antibiotics during the progress of DSS-induced colitis (n=5/group).

Data are representative of two (A) or three (B) independent experiments (mean±SEM in A-B). **p* < 0.05, ***p* < 0.01, ****p* < 0.001 by Student’s t test.

**References**

1. Song X, et al. Alterations in the microbiota drive interleukin-17C production from intestinal epithelial cells to promote tumorigenesis. *Immunity* **40**:140-52 (2014).

2. Hu S, et al. The DNA Sensor AIM2 Maintains Intestinal Homeostasis via Regulation of Epithelial Antimicrobial Host Defense. *Cell Rep* **13**:1922-36 (2015).

3. Ratsimandresy RA, Indramohan M, Dorfleutner A & Stehlik C. The AIM2 inflammasome is a central regulator of intestinal homeostasis through the IL-18/IL-22/STAT3 pathway. *Cell Mol Immunol* **14**:127-42 (2017).
